# Lineage hierarchies and stochasticity ensure the long-term maintenance of adult neural stem cells

**DOI:** 10.1126/sciadv.aaz5424

**Published:** 2020-04-29

**Authors:** Emmanuel Than-Trong, Bahareh Kiani, Nicolas Dray, Sara Ortica, Benjamin Simons, Steffen Rulands, Alessandro Alunni, Laure Bally-Cuif

**Affiliations:** 1 Zebrafish Neurogenetics Unit, Institut Pasteur, UMR3738, CNRS, Team supported by the Ligue Nationale Contre le Cancer, Paris 75015, France.; 2 Université Paris-Saclay, Ecole Doctorale Biosigne, Le Kremlin-Bicêtre, France.; 3 Max Planck Institute for the Physics of Complex Systems, Nöthnitzer Straße 38, 01187 Dresden, Germany.; 4 Department of Applied Mathematics and Theoretical Physics, Centre for Mathematical Sciences, University of Cambridge, Cambridge, UK.; 5 Wellcome Trust/Cancer Research UK Gurdon Institute, University of Cambridge, Cambridge UK.; 6 Wellcome Trust/Medical Research Council Stem Cell Institute, University of Cambridge, Cambridge UK.; 7 Center for Systems Biology Dresden, Pfotenhauer Str. 108, 01307 Dresden, Germany.

## Abstract

The cellular basis and extent of neural stem cell (NSC) self-renewal in adult vertebrates, and their heterogeneity, remain controversial. To explore the functional behavior and dynamics of individual NSCs, we combined genetic lineage tracing, quantitative clonal analysis, intravital imaging, and global population assessments in the adult zebrafish telencephalon. Our results are compatible with a model where adult neurogenesis is organized in a hierarchy in which a subpopulation of deeply quiescent reservoir NSCs with long-term self-renewal potential generate, through asymmetric divisions, a pool of operational NSCs activating more frequently and taking stochastic fates biased toward neuronal differentiation. Our data further suggest the existence of an additional, upstream, progenitor population that supports the continuous generation of new reservoir NSCs, thus contributing to their overall expansion. Hence, we propose that the dynamics of vertebrate neurogenesis relies on a hierarchical organization where growth, self-renewal, and neurogenic functions are segregated between different NSC types.

## Teaser

Adult neural stem cells in vertebrates are maintained at long term through a hierarchy of subfunctionalized stem cell pools.

## INTRODUCTION

The brain of most adult vertebrate species, including human, hosts specialized precursor cells, called neural stem cells (NSCs), which fuel the ongoing production of neurons into discrete brain regions (*1*–*5*). Adult-born neurons bring an additional layer of plasticity into local circuits that appears critical for particular aspects of learning and memory (*6*). In mammals, alterations of adult neurogenesis have been associated with Alzheimer’s disease and functionally linked to both major depression- and anxiety-like behaviors; it is thus of critical importance to understand the cellular and population rules that underlie NSC maintenance (*6*–*8*). However and despite extensive studies, the functional and molecular identity, long-term renewal potential, and pattern of division of NSCs remain controversial. Notably, while several works suggest that NSCs in both the dentate gyrus (DG) and the subependymal zone (SEZ) of the mammalian telencephalon are progressively consumed over time (*9*–*14*), others report a substantial self-renewal (*15*–*17*), or possibly amplification, potential of NSCs (*18*). Similar discrepancies were also reported in the adult zebrafish telencephalon (*19*, *20*). Accordingly, most models put forward in these studies also reached conflicting conclusions regarding the mode of NSC divisions. Whether these divergences reflect technical specificities and/or represent an underlying heterogeneity of the NSC compartment remains an important open question.

**Fig. 1:**
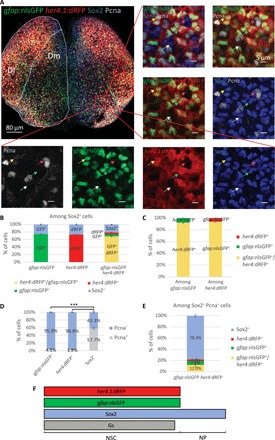
*her4.1*:dRFP and *gfap*:nlsGFP expressions characterize the same subpopulation of Sox2-expressing pallial progenitors. (**A**) Three-dimensional (3D) reconstruction (dorsal view) of the pallium of a *her4.1:dRFP/gfap:nlsGFP* double transgenic adult immunostained for GFP, dRFP, Sox2, and the proliferation marker proliferating cell nuclear antigen (Pcna) [labeling the same cells as the proliferation marker minichromosome maintenance 5 (*21*)]. The lateral and the medial pallial domains (Dl and Dm) are highlighted with dotted lines. Close-ups of the boxed area with different combinations of channels illustrating the four Sox2^+^ progenitor subtypes that make up the pallial germinal zone are shown: quiescent NSCs (qNSCs; blue arrow), activated NSCs (aNSCs; yellow arrow), qNPs (white arrow), and aNPs (pink arrow). (**B**) Distribution of *gfap^+^* and *her4.1^+^* NSCs, alone and in combination, among Sox2^+^ cells. (**C**) Respective distributions of *her4.1^+^* and *gfap*^+^ cells among the *gfap^+^* and the *her4.1^+^* populations. Paired *t* test: *P* = 0.34. (**D**) Relative proportions of quiescent (Pcna^−^) and proliferating (Pcna^+^) *gfap*^+^ NSCs, *her4.1*^+^ NSCs, and NPs (Sox2^+^). The data were analyzed using a repeated measures mixed model. Overall tests: *P* < 0.001; pairwise comparisons: ***P < 0.001 after Holm’s adjustment. (**E**) Distribution of *gfap^+^* and *her4.1^+^* NSCs together with Sox2^+^ NPs among proliferating (Pcna^+^) progenitors. (**F**) Summary of markers characterizing Dm pallial progenitors. (B to E) *n* = 7 brains were analyzed. Error bars, SEM.

To resolve the functional identity, fate behavior, and lineage dependencies of NSCs in an adult vertebrate brain, we used quantitative clonal analysis to target their fate in the zebrafish dorsal telencephalon (pallium). The everted morphology of the zebrafish pallium exposes its ventricle (Fig. 1A), making constituent NSCs and their progeny readily accessible to whole-mount investigation and allowing a combination of intravital imaging and genetic cell lineage tracing over a lifetime (*21*, *22*). Furthermore, under physiological conditions, progenitor cell migration is never observed and newly generated neurons delaminate to settle under the pallial ventricular surface adjacent to the place where they were born (*20*, *23*, *24*). Thus, the zebrafish pallium provides an optimal system to perform clonal analysis of NSC fates, as neural clones remain compact and superficial, allowing their whole cellular complement to be captured in a single in toto confocal acquisition.

Taking advantage of this unique system, we report that adult pallial NSCs are endowed with long-term self-renewal potential and are functionally heterogeneous. Notably, we provide evidence that adult NSCs are hierarchically organized into deeply quiescent and self-renewing “reservoir” NSCs (rNSCs) and downstream “operational” NSCs (oNSCs) supporting the bulk of neurogenesis. We further show that this NSC hierarchy is dominated by a previously unidentified pool of progenitors, responsible for the ongoing production of new rNSCs and the ensuing expansion of the entire NSC population. Last, we demonstrate that adult neurogenesis results overall in a net accumulation of adult-born neurons in the zebrafish pallium. These results comprehensively resolve the functional heterogeneity and fate behavior of NSCs in the adult vertebrate brain.

## RESULTS

### *her4.1* and *gfap* expressions identify the same population of progenitors, with largest coverage of astroglial cells

Zebrafish pallial NSCs share the same basic regulatory mechanisms and physiological requirements as mammalian NSCs (*25*). On the basis of markers, these radial glia-like astroglial cells are characterized by the expression of the glial fibrillary acidic protein (Gfap) and the enzyme glutamine synthetase (Gs), whose expression is redundant with Gfap, the Notch-target gene *hairy-related 4 tandem duplicate 1* (*her4.1*; orthologous to mammalian *Hes5*), and the stem cell–associated transcription factor Sox2 (sex determining region Y–box 2) (*5*, *21*, *26*–*29*). *her4.1*:dRFP (destabilized red fluorescent protein) and *gfap*:nGFP (nuclear green fluorescent protein) expressions in the pallium of double transgenic fish almost completely overlap and appear to characterize the same, mostly quiescent NSC population, which accounts for about 75% of Sox2-expressing cells (Fig. 1, A to D and F). The remaining Sox2^+^ cells express neither *her4.1* nor *gfap* and are, for the majority (57.7 ± 2.1%), in an activated/proliferating state [identifying them as activated non-astroglial neural progenitors (aNPs) against 42.3 ± 2.1% quiescent/nonproliferating NPs (qNPs)] (Fig. 1D). aNPs make up the bulk of actively proliferating pallial progenitors (Fig. 1, A and E), likely representing transit amplifying progenitors, which generate neurons after a limited number of cell divisions (*20*, *27*, *28*).

### Sparse genetic labeling of *her4.1^+^* NSCs allows the long-term clonal analysis of their fate

**Fig. 2:**
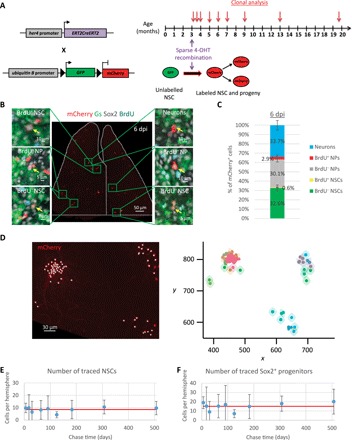
Lineage tracing of *her4.1*-expressing NSCs shows homeostasis. (**A**) Cre recombination was sparsely induced in 3-mpf *her4.1:ERT2CreERT2;ubi:Switch* double transgenic adults by 4-OHT, resulting in mCherry expression in recombined NSCs and their progeny. Analyzed time points (arrows) span between 6 and 507 dpi. (**B**) Dorsal view of a representative pallium showing sparsely induced cells at 6 dpi (dotted area to the pallial Dm territory of interest; fig. S1E). Boxed areas are magnified to illustrate the different cell types traced (yellow arrows). Proliferating progenitors were labeled by a 24-hour 5-bromo-2′-deoxyuridine (BrdU) pulse. NSCs are Gs^+^ and Sox2^+^, NPs are Sox2^+^ only. In contrast to aNSCs and aNPs, qNSCs and qNPs did not incorporate BrdU during the pulse. (**C**) Cell type composition of the mCherry^+^ population at 6 dpi. *n* = 6 brains. (**D**) Example of clustering at 183 dpi. Left: Dorsal view of a 3D reconstruction of a hemisphere. The spots registering the coordinates of traced cells (red) are shown (white dots). Right: 2D map of the highest probability clonal partition identified by the statistical inference. Axes correspond to the *x* and *y* coordinates (in μm) of the 2D projection of the cell centers. Cells belonging to the same clone are embedded in the same shaded area. (**E**) Average number of traced (mCherry^+^) Gs^+^/Sox2^+^ NSCs per hemisphere across all time points analyzed. One-way analysis of variance (ANOVA): *F*_(8,37)_ = 1, *P* = 0.45; all pairwise comparisons: least significant difference (LSD) test followed by Holm’s adjustment. Error bars, 95% confidence interval (CI). (**F**) Average number of traced (mCherry^+^) Sox2^+^ cells per hemisphere across all time points analyzed. One-way ANOVA: *F*_(8,37)_ = 1.65, *P* = 0.14; all pairwise comparisons: LSD test followed by Holm’s adjustment. Error bars, 95% CI. (E and F) *n* = 6, 3, 3, 3, 4, 6, 7, 8, and 6 brains at 6, 18, 30, 64, 91, 125, 183, 307, and 507 dpi, respectively.

To target the fate of individual pallial NSCs and their progeny in the dorsomedial pallial domain (Dm) during adulthood over extended periods of time, we opted for an inducible genetic lineage tracing approach (Fig. 2A). We crossed the transgenic driver line *Tg(her4.1:ERT2CreERT2)* (referred to as *her4.1:ERT2CreERT2*), which expresses a tamoxifen-inducible Cre recombinase in *her4.1*-expressing NSCs (fig. S1, A and B), with the *Tg(-3.5ubb:loxP-EGFP-loxP-mCherry)* (for short, *ubi:Switch*) reporter line (*30*). Overall, in the analyzed region (see below), *her4.1:ERT2CreERT2;ubi:Switch* fish display neglectable levels of uninduced recombination: We recovered only six cells, all in a single hemisphere, from a total of 10 brains from noninduced double transgenic fish, at an age [14-months post-fertilization (mpf)] covering most of the time span of the clonal analysis (fig. S1, C and D). To establish the conditions for clonal induction, we induced 3-mpf adult zebrafish with decreasing concentrations and exposure times to 4-hydroxytamoxifen (4-OHT) until reaching an average number of 20.7 ± 2.45 (means ± SEM) labeled cells (or cell clusters) per hemisphere at 6 days post-induction (dpi) (Fig. 2, A to C, and fig. S4E). The slow accumulation of mCherry protein precluded the reliable counting of labeled cells at earlier time point, and by 6 dpi, 46% of the clones had already divided and/or differentiated into neurons (fig. S2). However, by that time, only 1.6 ± 0.3% of the total Sox2^+^ cell population was marked with mCherry (fig. S9E). Analysis of nearest-neighbor distances (NNDs) between the centers of Sox2^+^ clones identified visually indicated that their majority lies at a distance greater than 34 μm from each other, i.e., about eight NSC diameters (figs. S4, A and B, and S9B) (except in the most posterior part the pallium, which was therefore excluded from the analysis on the basis of anatomical landmarks; fig. S1E). In line with the very low rate of apoptosis during adult pallial neurogenesis in zebrafish (*20*), we detected only a few cells displaying caspase-3 activity in the analyzed region (means ± SEM: 7.8 ± 1.8) (fig. S4, C and D). Thus, besides the sparse labeling of cells following induction, the clonality of the data is also suggested by the roughly invariable average number of clones across all the time points analyzed (with the exception of the latest at 507 days; see Supplementary Materials and Methods) (fig. S4, E to G). This result is in line with an absence of clonal fusion or fragmentation.

However, to more rigorously identify the most likely clonal partition of cells while at the same time quantifying the amount of uncertainty stemming both from the clustering process and from clonal ambiguities (*31*), we also combined statistical inference with a biophysical model of clone dispersion. Specifically, for each sample, we partitioned labeled cells into putative clones and, taking into account the dynamics of clone dispersion, calculated the probability that the centers of these clones are in agreement with statistically independent induction events. We took the partition with the highest probability, as well as all partitions within uncertainty bounds, for further analysis (Fig. 2D and fig. S4, H to J; see also Supplementary Theory). Together, this approach allowed us to perform clonal analysis while accounting for the potential uncertainty of clonal assignments.

### *her4.1^+^* NSCs are endowed with long-term self-renewal potential

Stem cells are defined functionally by their long-term self-renewal capacity. Following the report that zebrafish pallial NSCs might be progressively consumed through direct differentiation into neurons (*19*), in apparent contradiction with their measured steady density over the considered time frame (*32*), we first explored their maintenance within the *her4.1* lineage. Notably, we found that the total number of *her4.1:ERT2CreERT2*-traced Gs^+^;Sox2^+^ NSCs, averaged across animals, was maintained at a seemingly constant level throughout the ~17-month chase period spanning more than half the adult zebrafish life (Fig. 2E). This result also applied to the whole population of traced Sox2^+^ progenitors (Fig. 2F). Thus, the targeted *her4.1* lineage is, as a whole, homeostatic and self-reliant for its maintenance, a behavior consistent with asymmetric fate in which the size of the labeled NSC pool remains constant over time.

### *her4.1^+^* NSCs are heterogeneous and organized along a strict hierarchy

This fate asymmetry can be achieved either at the level of each and every NSC division or at the level of the population. Therefore, to gain insight into the fate of individual NSCs, we monitored the behavior and composition of *her4.1^+^* NSC-derived clones over the ~17-month chase period. We first noted that NSCs (Gs^+^;Sox2^+^) maintained an overall fixed ratio to NPs (Gs^−^;Sox2^+^), whether considering the whole population of pallial progenitors or the *her4.1* lineage (fig. S5). These observations suggest that the behavior of the entire population of Sox2^+^ pallial progenitors is slave to that of the renewing NSC population. Therefore, since Sox2 nuclear localization improves the reliability of cell identity assignment, Sox2 expression was used as a surrogate NSC marker.

**Fig. 3:**
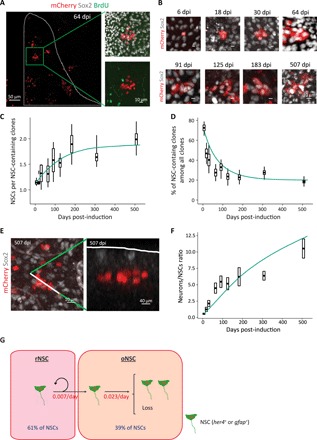
Hierarchical organization of pallial NSCs. (**A**) Dorsal view of a pallial hemisphere exemplifying some clones recovered at 64 dpi (magnification of the boxed area shows one clone). The analyzed region is outlined with dotted lines. (**B**) 3D reconstruction of clones at different time points illustrating the progressive increase in their NSC content until 183 dpi. (**C**) Time evolution of the number of NSCs (i.e., Sox2^+^ cells) per NSC-containing clone. (**D**) Time evolution of the proportion of NSC-containing clones among all clones. (**E**) Left: 3D reconstruction of a multicelled fully neuronal clone at 507 dpi. Right: Optical section along the plane defined by the dotted line on the 3D reconstruction (left). Note that the clone does not contain NSCs anymore and is completely detached from the ventricular zone (dotted line). (**F**) Time evolution of the ratio of neurons to NSCs within the entire population of traced cells. (**G**) Schematic illustrating the simplified model used for maximum likelihood estimation. The inferred activation frequency and relative proportions of both rNSCs and oNSCs are given in red and blue, respectively (rNSC ➔ rNSC + oNSC, ν = 0.007 day^−1^; oNSC ➔ oNSC + oNSC, λ = 0.006 day^−1^; oNSC ➔ loss, μ = 0.017 day^−1^). (C and D) Box-and-whisker plots, experimental data. The central bold bar and the upper and lower edges of the boxes represent the means and SEM of the most likely clonal composition, respectively; the whiskers of the box respectively correspond to the 95% CIs of the most extreme clonal assignments still in agreement with clonality (see Supplementary Materials). They reflect the combined uncertainty stemming from the clonal reconstruction and the finite sample size. (C, D, and F) Clones as defined by the algorithm. *n* = 6, 3, 3, 3, 4, 6, 7, 9, and 7 brains at 6, 18, 30, 64, 91, 125, 183, 307, and 507 dpi, respectively. Solid lines depict modeling predictions.

Consistent with a population asymmetry-based model of NSC self-renewal, we found that the fraction of clones containing at least one NSC (hereafter referred to as “NSC-containing clones”) decreased continuously over the first several months after induction and was matched by a concomitant increase in the average number of NSCs per NSC-containing clone (Fig. 3, A to E, and fig. S6, A and B). However, by 183 dpi, both measures reached a plateau that persisted until the end of the analysis. The emergence of a plateau in clone statistics stands in contrast to that expected for a continuous process of NSC loss and replacement, where “neutral drift” would result in both an ever-decreasing number of NSC-containing clones and an ever-increasing number of NSCs in these clones (*33*). Instead, stationarity in both the proportion and the size of NSC-containing clones, as observed here at long chase times, is a behavior typically expected from a population of NSCs in which long-time renewal potential relies on asymmetric fate at the level of individual cell divisions.

The different dynamics observed before and after 183 dpi could reflect a change in NSC behavior with age. Arguing against this interpretation, however, we found that the size of the traced NSC population, the proliferative activity of NSCs, and their fate choices at division remained approximately constant over the chase period (Fig. 2, E and F, and figs. S6, C and D, and S8D). Thus, the apparent crossover in clone behavior is not associated with a change in developmental dynamics. As an alternative, we therefore considered that two different, hierarchically related subpopulations of NSCs might have been targeted upon induction: an upstream subpopulation of asymmetrically dividing NSCs, the behavior of which is revealed upon long chase times, and another downstream subpopulation, which dominates clonal dynamics at earlier chase times and whose NSCs undergo population asymmetry. The plateauing in the number of NSCs per NSC-containing clones also suggests that this downstream subpopulation displays limited self-renewing potential, as it indicates that its initially labeled NSCs ultimately became exhausted (balanced fates would have resulted in a continuous increase in the number of NSCs per NSC-containing clones). This conclusion is further supported by the greater loss rate of NSC-containing clones as compared to their rate of expansion (Fig. 3, C and D, and fig. S6, A and B).

### Modeling supports a hierarchical organization in which rNSCs divide asymmetrically to give rise to oNSCs adopting unbalanced stochastic fates

Thus, we hypothesized that *her4.1*-expressing NSCs were organized in a proliferative hierarchy, with a self-renewing subpopulation of rNSCs supporting a second population of oNSCs with limited neurogenic potential. To test whether such a scheme could describe quantitatively the clonal data, we developed a minimal model. Within this framework, rNSCs divide asymmetrically at rate ν, giving rise to oNSCs. To capture the variability of clonal outputs, we propose that oNSCs follow a pattern of stochastic fate, duplicating at rate λ and becoming lost through differentiation at rate μ (Fig. 3G). (Later, we consider a more refined model that takes into account the whole set of actual oNSC fates.) A fit to the data using maximum likelihood estimation indicated that rNSCs would constitute some 61% of NSCs and enter into cycle around once every 143 days, on average, while oNSCs (forming the remaining 39% of NSCs) would select between duplication or loss once every 43 days in the ratio of 1 to 3, respectively [for details and statistical confidence intervals (CIs), see Fig. 3G, fig. S7A, and Supplementary Theory]. In addition to the average NSC content and proportion of NSC-containing clones (Fig. 3, C and D), the model was also able to predict the detailed clone size distributions over the entire time course (fig. S7, B and C). Although NSCs and NPs were pooled to increase statistical confidence, quantitatively similar results were obtained when the analysis was performed on the Gs^+^ compartment alone.

The proportions of rNSCs and oNSCs predicted by the model (61 and 39%, respectively) appeared different from the ones suggested by the experimental data. From the proportion of NSC-containing clones at the plateauing chase time, it can be estimated that some 20% of the initially labeled NSCs belong to the reservoir pool (against 80% for the operational pool) (Fig. 3D and fig. S6B). This discrepancy likely stems from the biased recombination of the *ubi:Switch* reporter upon clonal induction, favoring oNSCs. The high proportion of traced cells that had already activated by 6 dpi (fig. S2) is in line with this interpretation, suggesting that induction was biased toward the more active pool of oNSCs. Hence, while the initially targeted *her4.1^+^* NSC population actually comprised 20% rNSCs/80% oNSCs, its NSC makeup progressively changed over time to reach (around 183 dpi) its steady-state composition (61% rNSCs/39% oNSCs), as oNSC clones became lost and new oNSCs arose from rNSCs in the remaining NSC-containing clones (fig. S7A).

### Intravital imaging reveals that NSCs take overall balanced patterns of fates

**Fig. 4:**
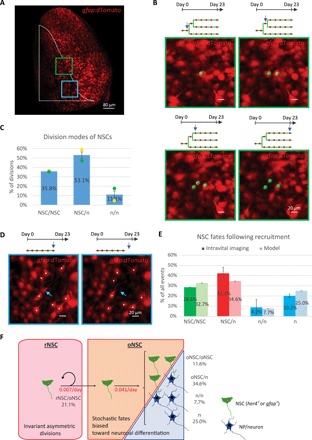
Time-lapse intravital imaging analysis of NSC division patterns. (**A**) Dorsal view (3D reconstruction) of a pallial hemisphere imaged from a live *casper;gfap:dTomato* fish. The region analyzed is surrounded by the dotted line. The green and blue squared areas are enlarged in (B) and (D), respectively. (**B**) Example of an NSC that underwent two symmetric amplifying divisions. Green spots are superimposed to the NSCs of interest; tracks (green) with red dots indicating imaging sessions where the NSCs were still present (blue arrows point to the moments of image acquisition). (**C**) Relative proportions of the different types of NSC divisions inferred from their tracking in live fish (color coded). n, loss of *gfap* expression (NP/future neuron). *n* = 2 brains. (**D**) Example of a direct differentiation of an NSC (loss of *gfap* expression) (blue arrows). Asterisks (*) mark surrounding NSCs as visual landmarks. (**E**) Relative proportions of NSC fates leading to expansion (symmetric amplifying divisions; green), maintenance (asymmetric divisions; red), and consumption (symmetric neurogenic divisions and direct neuronal differentiations; blue). Light colors are the results of our simulations. Note the overall balance in NSC fates. *n* = 2 brains. Error bars, SD and credibility interval (see Supplementary Theory) for the in vivo data and the modeling, respectively. (**F**) Modeling of NSC dynamics. The inferred proportions and frequencies (in red, values per cell) of both rNSC and oNSC fates upon activation are indicated (oNSC ➔ oNSC + n, κ = 0.018 day^−1^; oNSC ➔ n + n, μ_1_ = 0.004 day^−1^; oNSC ➔ n, μ_2_ = 0.013 day^−1^). The corresponding curves are plotted in green above experimental data in Fig. 3 (C, D, and F). Note that NSC/NSC fates monitored by live imaging include both rNSC/oNSC and oNSC/oNSC divisions.

To further test the basis of this hierarchical scheme and dissect in detail the fate behaviors of NSCs, we took advantage of our intravital imaging method, which permits individual NSCs to be recorded in their niche, tracking >300 NSCs per hemisphere in the Dm over several weeks under fully noninvasive conditions (*21*). *gfap:dTomato* transgenic individuals were crossed into the transparent *casper* background and imaged live over 23 days (Fig. 4A) (*21*, *34*). We first determined the time needed for NSC fates to become apparent. For this, we focused on symmetric neurogenic divisions, as an unambiguous fate accessible during the time span of our experiment. Analysis of the time elapsed between an NSC division and the longest time needed by daughter cells to lose *gfap*:dTomato expression, revealed that ~75% of such neurogenic fates become resolved by 10 days following division. (fig. S8, A and B). Consequently, tracks with less than 10 days available after division were not included in the analysis (fig. S8C). We did not detect caspase-3 activity in any ventricular pallial progenitor, ruling out that loss of *gfap* expression could result from apoptosis (fig. S4, C and D).

Hence, by analyzing an average cohort of 25 ± 4 (means ± SEM) active tracks per fish (i.e., NSCs that became activated during the time frame of analysis), we found that 35.8 ± 0.7% of NSC divisions result in symmetric amplifying fate (NSC/NSC fate, as inferred from *Gfap* expression), 53.1 ± 2.5% result in asymmetric fate (NSC/n, where “n” is a neuron or an NP, i.e., a cell that lost *gfap* expression), and 11.1 ± 1.6% result in symmetric differentiating fate (n/n) (Fig. 4, B and C). Similar statistics were inferred from the analysis of two-cell clones recovered at 6 dpi, with values that were not altered with age (fig. S8D). Notably, both intravital imaging and inspection of single-celled clones at 6 dpi revealed the occurrence of direct (i.e., without cell division) differentiation of NSCs into neurons (Fig. 4D and fig. S8, C and E) (*19*). In line with the homeostatic nature of NSC dynamics within the *her4.1* lineage (Fig. 2, E and F), we found that direct neuronal differentiations accounted for about 20.2 ± 1.7% of all fate choice events, thus contributing significantly to the overall balance in fates (Fig. 4E) (*19*, *21*).

### Modeling can independently predict the NSC fates inferred from intravital imaging

To comprehensively model the *her4.1* lineage, we then introduced the different qualitative fates observed by intravital imaging (Fig. 4, A to E) into the previously determined dynamical parameters of rNSCs and oNSCs (Fig. 3G) and inferred the predicted rates of both asymmetrical (κ) and symmetric neurogenic divisions (μ_1_) of oNSCs. This allowed us to provide a complete description of NSC dynamics, including the rates of all fate transitions for oNSCs (Fig. 4F). As an important validation, we found the quantitative pattern of NSC fates predicted by our model to be in good agreement with the measurements scored in live fish (Fig. 4E) [“NSC/NSC” fates include the divisions of rNSCs, which are asymmetric and generate rNSC/oNSC fate, and the amplifying divisions of oNSCs (oNSC/oNSC fate), as they are indistinguishable in live imaging]. Furthermore, the derived frequencies of all NSC divisions (rNSCs and oNSCs) add up to 0.035 per day, a figure commensurate with the measured division rate of pallial NSCs (Fig. 1D and fig. S6C).

Overall, these results are compatible with a model where pallial NSCs are heterogeneous and organized in a hierarchy in which an upstream reservoir population divides asymmetrically, ensuring lineage homeostasis, and infrequently to produce oNSCs with only limited renewal potential. The latter, despite being also mostly quiescent, activate more frequently and follow a pattern of stochastic fate biased toward neuronal differentiation, thus comprising the effective neurogenic pool.

### A previously unrecognized progenitor source fuels the ongoing production of new NSCs

**Fig. 5:**
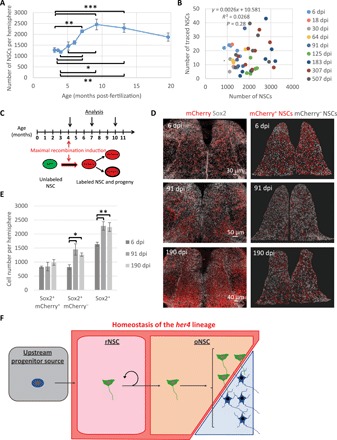
Ongoing production of *her4^+^* NSCs by an upstream progenitor source. (**A**) Quantification of the total number of NSCs (as assessed by Sox2 expression) per hemisphere in the Dm territory of interest. One-way ANOVA: *F*_(8,37)_ = 8.06, *P* < 0.001; all pairwise comparisons: LSD test followed by Holm’s adjustment. *P < 0.05, **P < 0.01, and ***P < 0.001. *n* = 6, 3, 3, 3, 4, 6, 7, 8, and 6 brains at 3.1, 3.6, 4, 5.1, 6, 7.1, 9.2, 13.2, and 19.6 mpf, respectively. Error bars, SEM. (**B**) Scatter plot showing the absence of correlation between the total numbers of NSCs (Sox2^+^ cells) in the pallial region analyzed and the number of NSCs (Sox2^+^ cells) in the traced *her4.1* lineage. *n* = 6, 3, 3, 3, 4, 6, 7, 8, and 6 brains at 6, 18, 30, 64, 91, 125, 183, 307, and 507 dpi, respectively. Cell numbers are per hemisphere. (**C**) Timeline of the experiment. Maximal recombination of the *ubi:Switch* reporter was induced by treating *her4.1:ERT2CreERT2;ubi:Switch* transgenic fish with 4-OHT repetitively over 5 days. (**D**) Left: Dorsal view of pallia immunostained for mCherry and Sox2 at 6, 91, and 190 dpi. Right: Segmented Sox2^+^ NSCs (spots). Red spots, NSCs within the *her4.1* lineage (Sox2^+^ and mCherry^+^); white spots, mostly newly formed NSCs (Sox2^+^ and mCherry^−^). (**E**) Quantification of Sox2^+^/mCherry^+^, Sox2^+^/mCherry^−^, and Sox2^+^ cells in the Dm territory of interest (numbers of cells are per hemisphere). One-way ANOVA: Sox2^+^/mCherry^+^ cells, *F*_(2,13)_ = 0.59 and *P* = 0.5697; Sox2^+^/mCherry^−^ cells, *F*_(2,13)_ = 6.02 and *P* = 0.0141; Sox2^+^ cells, *F*_(2,13)_ = 9.06 and *P* = 0.0034. All pairwise comparisons: LSD test followed by Holm’s adjustment. *P < 0.05 and **P < 0.01. Error bars, SEM. *n* = 5, 6, and 5 brains at 6, 91, and 190 dpi, respectively. (**F**) Proposed hierarchical organization of pallial NPs.

The maintenance of a relatively constant number of NSCs within the *her4.1* lineage was unexpected (see also Supplementary Materials and Methods). Since the zebrafish brain continues to grow during adulthood (*32*), expansion of the NSC pool has been long hypothesized to accompany the ongoing growth of the pallium. Accordingly, we found that the total number of pallial NSCs expanded roughly linearly with time until 9 months of age (Fig. 5A and fig. S9A). Analysis of NNDs revealed no significant change in NSC density over the same time frame, as well as no correlation between NSC numbers and densities, suggesting that the ventricular area progressively enlarges as the brain and the NSC pool grow (fig. S9, B and C). Variation in labeling efficiency did not blur a potential increase in the traced NSC pool, as we found no correlation between the number of *her4.1:ERT2CreERT2*-traced NSCs and the total number of NSCs and only a poor and nonrelevant correlation between the total number of NSCs and the NSC content of NSC-containing clones (Fig. 5B and fig. S9D). Furthermore, during the growth period, the *her4.1:ERT2CreERT2*-derived pool of NSCs tended to represent an ever-decreasing fraction of the total NSC population (fig. S9E), as expected for a homeostatic pool evolving in a continuously growing population. As *her4.1*^+^ and Sox2^+^ cells maintained a fixed ratio with time, it follows that the overall population of *her4.1*^+^ NSCs continued to expand (Fig. 5A and fig. S9, F and G).

Together, these results suggest that new *her4.1*^+^ NSCs are generated over time from initially unlabeled cells, i.e., which do not belong to the *her4.1^+^* lineage, as targeted by the clonal induction. To add direct support to this interpretation, we devised a protocol for maximal induction with the *her4.1:ERT2CreERT2* driver line (Fig. 5C), initially labeling around 50% of Sox2^+^ cells (versus ~70% for *her4.1*^+^ cells; Figs. 5, D and E, and 1B). Quantification of the traced (mCherry^+^) NSC bulk population confirmed the invariance of the number of NSCs in the *her4.1*^+^ lineage (Figs. 5, D and E, and 2, E and F). In marked contrast, quantification of unlabeled (mCherry^−^) NSCs revealed a noticeable expansion of their population during the chase time (Fig. 5, D and E), suggesting that new NSCs are continuously produced within the pallial niche. Hence, our results point to the existence of a yet undefined “NSC source,” hierarchically upstream of *her4.1*^+^ NSCs and responsible for their continuing production over time. The dynamics of the whole NSC population also predicts that the activity of the source fades after 9 to 10 mpf (Fig. 5A).

### Pallial adult neurogenesis is additive

**Fig. 6:**
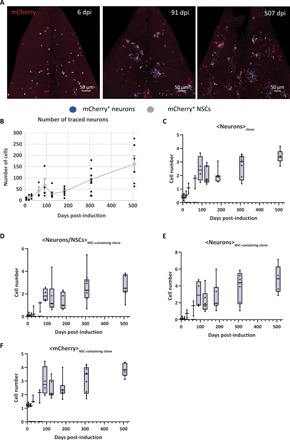
Pallial adult neurogenesis is additive in zebrafish. (**A**) Dorsal views of the analyzed pallial Dm region at different time points of the clonal analysis. Only the mCherry channel is displayed to highlight the clones. NSCs and neurons are marked by white and blue spots, respectively. (**B**) Evolution over time of the average number of traced neurons. One-way ANOVA: *F*_(8,37)_ = 10.12, *P* < 0.001; all pairwise comparisons: LSD test followed by Holm’s adjustment (the results of pairwise comparisons are given in data file S1). Error bars, SEM. *n* = 6, 3, 3, 3, 4, 6, 7, 8, and 6 brains at 6, 18, 30, 64, 91, 125, 183, 307, and 507 dpi, respectively. (**C**) Time evolution of the average number of neurons per clones. (**D**) Evolution over time of the ratio of neurons to NSCs in the NSC-containing clones. (**E**) Evolution of the number of neurons per NSC-containing clone across all time points analyzed. (**F**) Evolution over time of the average clone size. (C to F) Box-and-whisker plot: The horizontal bar indicates the median, the lower and upper edges of the box represent the first and third quartiles, respectively. The whiskers extend to the most extreme data point. Kruskal-Wallis test: *P* < 0.0001; all pairwise comparisons: Behrens-Fisher tests (for the sake of the graphs readability, the results of pairwise comparisons are given in data file S1). (C to G) *n* = 6, 3, 3, 3, 4, 6, 7, 9, and 7 brains at 6, 18, 30, 64, 91, 125, 183, 307, and 507 dpi, respectively.

Last, we took advantage of the clonal data to gain insight into the production dynamics of new neurons. The total number of labeled neurons progressively increased over the full extent of the experiment which, with regard to the constant number of traced NSCs, indicates that adult-born neurons accumulate over time (Figs. 2, E and F; 5E; and 6, A and B). In agreement with the near absence of apoptosis during adult neurogenesis in this pallial region (*20*), our quantitative model also provided a very good prediction of the time increase in the ratio of neurons to NSCs in the lineage (Fig. 3F). These results also applied at the level of individual clones (Fig. 6, C to E). Notably, the continuous increase of the ratio of neurons to NSCs in NSC-containing clones underscores that the main driver of clonal growth is the generation of new neurons (Fig. 6, D to F). Furthermore, the lack of a major inflection in the time evolution of this ratio provided additional evidence that the plateaus observed in the clonal NSC statistics were not linked to an increase in NSC quiescence. Thus, similarly to the rodent DG (*35*), we found that zebrafish adult pallial neurogenesis is additive, i.e., results in a net addition of new functional neurons with time (*20*).

## DISCUSSION

Understanding the functional behavior and hierarchical organization of NSCs is a prerequisite to defining their molecular identity, regulation, evolution with age, and their response to challenge. By integrating genetic tracing, intravital imaging, and global population assessments at short and long term over a lifetime, our results are broadly compatible with a model where, in the adult zebrafish pallium, deeply quiescent self-renewing NSCs (termed rNSCs) reside at the apex of a proliferative hierarchy and divide asymmetrically to give rise to other slow-cycling NSCs (termed oNSCs), harboring a higher activation frequency, endowed with only limited renewal potential and characterized by stochastic fates. They further suggest that, above this hierarchy, an as yet uncharacterized progenitor source fuels the continuous production of an intrinsically homeostatic population of NSCs, hence contributing to its overall expansion (Fig. 5F). Last, we bring evidence that adult pallial neurogenesis is additive, i.e., that neurons generated during adulthood tend to accumulate instead of simply contributing turnover.

By providing a complete model of NSC maintenance in an adult vertebrate brain based on comprehensive quantitative dynamics, this study supports, first, that common molecular and cellular signatures of NSCs (*her4.1* and *gfap* expression) mask functional heterogeneities and, second, that NSC dynamics involve a hierarchy of specialized subpopulations, comprising bona fide self-renewing NSCs (reservoir) supporting a shorter-lived (operational) neurogenic pool. As a result, this model may reconcile previous phenotypical or lineage work on mammalian adult NSC pools.

A partition of NSCs between a mostly dormant and a more active pool (in which NSCs shuttle between rest and activation) was recently proposed in the mouse DG, based on phenotypical analysis of HUWE1 mutants, affecting the return of NSCs into quiescence upon division (*36*). Likewise, *Hes5*-expressing DG NSCs and Troy-expressing SEZ NSCs also appear to conform to a homeostatic behavior (*15*, *37*), suggesting that the previously reported activity-dependent consumption of NSCs might rather reflect the dynamics of an operational-like, or an activated, pool (*9*, *10*, *12*, *13*, *38*). Accordingly, all but one of the aforementioned studies supporting a “disposable stem cell” model implemented strategies likely to overrepresent frequently activating NSCs, such as 5-bromo-2′-deoxyuridine (BrdU) labeling (*10*, *12*), retrovirus-based lineage tracing (*12*), or *Ascl1*-CreERT2–based lineage tracing (*13*, *38*). These could correspond to a subset of oNSCs or to yet further committed progenitors.

Last, GFAP^+^ cells with radial morphology, potentially NSCs, were also shown to increase in number over time within the *Nestin*:CreERT2-driven lineage in the DG (*18*), suggesting that a source population (presumably *Nestin^+^/Hes5^−^*) responsible for NSC production might also exist in the mouse brain. Currently, the absence of specific markers for source progenitors hampers their proper characterization. Determining their precise abundance, division properties, self-renewal potential, and origin will be essential steps toward a better understanding of the mechanism underlying NSC population dynamics. Thus, while the model that we propose primarily applies to the adult pallial neurogenic area of the zebrafish brain, potentially reconciling apparently conflicting results (*19*, *20*), it may provide an interesting framework to rethink and explore further NSC self-renewal and diversity in other systems, such as the mammalian brain. It also suggests that adult germinal populations that increase in cell number during adult life may combine homeostatic dynamics typical of mature tissues with a generator (“source”) akin to embryonic growth zones.

## MATERIALS AND METHODS

### Zebrafish care and strains

All procedures relating to zebrafish (*Danio rerio*) care and treatment conformed to the directive 2010/63/EU of the European Parliament and of the council of the European Union. Zebrafish were kept in 3.5-liter tanks at a maximal density of five per litter, in 28.5°C and pH 7.4 water. They were maintained on a 14-hours light/10-hours dark cycle (light was on from 8 a.m. to 10 p.m.) and fed three times a day with rotifers until 14 days post-fertilization and with standard commercial dry food (GEMMA Micro from Skretting) afterwards. All transgenic lines—*Tg(her4.1:dRFP)* (*39*), *Tg(gfap:nGFP)^mi2004^* (*40*), *Tg(gfap:eGFP)^mi2001^* (*41*), *Tg(her4.1:ERT2CreERT2)* (*42*), and *Tg(-3.5ubb:loxP-EGFP-loxP-mCherry)* (referred to as *ubi:Switch)* (*43*)—were maintained on an AB background. The *Tg(*gfap:*dTomato)* (*44*) transgenic fish were kept on a *Casper* (*roy^−/−^;nacre^−/−^*) background (*34*). *Tg(her4.1:dRFP)*/+ fish were interbred with *Tg(gfap:nGFP)/+* fish to obtain *Tg(her4.1:dRFP)*/+; *Tg(gfap:nGFP)/+* double transgenic fish. Similarly, crosses between *Tg(her4.1:ERT2CreERT2)/+* and *Tg(-3.5ubb:loxP-EGFP-loxP-mCherry)/+* zebrafish yielded the *her4.1:ERT2CreERT2; ubi:Switch* double transgenic individuals used for the lineage tracing experiments. Ages of the fish are explicitly stated in the respective experiments (see also the “4-OHT treatment” section), except for the *Tg(her4.1:dRFP)*/+; *Tg(gfap:nGFP)/+* and *Tg(her4.1:ERT2CreERT2)/+; Tg(her4.1:dRFP)*/+ double transgenic fish as well as the Casper *Tg(gfap:dTomato)* fish, which were all 4 mpf. In addition, *Tg(gfap:eGFP)* (fig. S3, C and D) were 5 months old. Fish were euthanized in ice-cold water (temperature comprised between 1° and 2°C) for 10 min, according to a special dispensation and following the guidelines of the Ministry of Superior Education, Research, and Innovation.

### Genotyping

Fish were screened for the expression of the *her4.1:dRFP*, the *gfap:nGFP*, and the *ubi:Switch* transgenes between 48 and 72 hours post-fertilization. The presence of the *her4.1:ERT2CreERT2* transgene was detected by polymerase chain reaction (PCR) amplification of a part of the ERT2 sequence on a tail DNA sample. DNA was extracted with the “Phire Animal Tissue Direct PCR Kit” (Thermo Scientific) according to the manufacturer’s instructions, and PCR was performed with the following primers (*42*): forward, 5′-GACCCTCCATGATCAGGTCCACC-3′ and reverse, 5′-GACCGTGGCAGGGAAACCCTCTG-3′. The thermocycling parameters are found in Table 1. The resulting 676-base pair amplicon was resolved on a 2% agarose gel containing 0.002% SYBR Safe (Thermo Fisher Scientific, S33102).

**Table 1:** Thermocycling parameters used for the PCR amplification of the ERT2 fragment.

**Cycle step**	**Temperature**	**Time**	**Cycles**
Initial denaturation	94°C	2 min	1
Denaturation	94°C	15 s	30
Annealing	58.5°C	45 s	
Extension	72°C	45 s	
Final extension	72°C	1 min	1
	4°C	Hold	

### 4-OHT treatment

*her4.1:ERT2CreERT2; ubi:Switch* double transgenic fish were immersed either for 10 min in fish water containing 0.5 μM 4-OHT (Sigma-Aldrich, T176) for clonal recombination of the reporter construct or for 7 hours per day, for five consecutive days, in fish water containing 2 μM 4-OHT for maximal recombination. Practically, fish were placed in beakers containing 4-OHT dissolved in fish water and protected from light with foil. We used 25 ml of solution per fish for the 10-min induction protocol and 100 ml per fish for the full induction protocol. For the latter, fish were transferred to fresh water every night and fed in the morning before being returned to 4-OHT–containing water; water and 4-OHT solution were continuously oxygenated through a pump-generated air flow, and the 4-OHT solution was renewed every day. In both protocols, fish were rinsed at least three times with fresh water over 48 hours before being taken back to the fish facility. All fish used for the clonal analysis were 3 to 3.5 mpf at the time of induction. The analysis of two-celled clones (doublets of induced cells) in young and old fish was performed 6 days after induction in 3- and 14-mpf fish, respectively. The full induction experiment was carried out with 4-mpf fish.

### BrdU treatment

Proliferating progenitors of both *her4.1:dRFP* and clonally induced *her4.1:ERT2CreERT2; ubi:Switch* fish were labeled by a 24-hour BrdU pulse, just before being euthanized. For this purpose, fish were placed into fish water containing 1 mM BrdU and 0.333% (v/v) of dimethyl sulfoxide (DMSO; Thermo Scientific, 20688). Treatments were performed in beakers, with a maximum of 10 fish per liter of solution, under continuous air flow and at a temperature of 28°C. Fish were rinsed two times for 5 min in fresh water before euthanasia and dissection.

### Anesthesia (live imaging)

Anesthesia was initiated by soaking the fish for approximatively 90 s in water containing 0.01% MS222 (Sigma-Aldrich). They were then transferred into a water solution of 0.005% (v/v) MS222 and 0.005% (v/v) isoflurane to maintain the anesthesia during the whole duration of the imaging session (*21*). Overall, fish were anesthetized for about 30 min per session.

### Histology

Brains were dissected in phosphate-buffered saline (PBS; Fisher Bioreagents) and directly transferred to a 4% paraformaldehyde solution in PBS for fixation. They were fixed either for 2 hours at room temperature or overnight at 4°C under permanent agitation. After two washing steps in PBS, brains were dehydrated through a series of 25, 50, and 75% methanol in PBS and kept in 100% methanol (Sigma-Aldrich, 322415) at −20°C. Following rehydration, brains were processed for whole-mount immunohistochemistry. An antigen retrieval step was performed for subsequent BrdU and/or proliferating cell nuclear antigen (Pcna) immunolabeling. For BrdU, brains were incubated in 2 M HCl (Sigma-Aldrich, 258148) at room temperature for 30 min, whereas for Pcna immunolabeling, they were incubated with HistoVT One (Nacalai Tesque) for an hour at 65°C. Brains were rinsed three times for at least 5 min in PBS and then blocked with 4% normal goat serum, 0.1% DMSO, and 0.1% Triton X-100 (Sigma Life Science, 1002135493) in PBS (blocking buffer). They were then incubated at room temperature for 24 hours in primary antibodies (Table 2) diluted in blocking buffer, rinsed three times over 24 hours at room temperature with 0.1% Tween 20 (Sigma Life Science, P9416) in PBS (PBT), and incubated in secondary antibodies (Table 3) for another 24 hours at room temperature. After at least three rinses in PBT over 24 hours, brains were transferred into PBS, and their telencephala were dissected out. Every washing and incubation step was performed on a rocking platform and, from the secondary antibodies onwards, was carried out protected from light. Dissected telencephala were mounted in depression slides with Aqua-Poly/Mount (Polysciences, 18606). The brain sections presented in fig. S3 were processed similarly, with the following modifications. After rehydration, brains were embedded in 3% agarose, sliced in 50-μm-thick sections with a vibratome (Leica, VT1000 S) and recovered in PBS. Sections were incubated with primary and secondary antibodies overnight at 4°C and rinsed three times over the day in PBT. Last, sections were incubated for 10 min in 4′,6-diamidino-2-phenylindole (1 μg/ml) in PBS, rinsed three times for 5 min in PBS, and mounted on slides with Aqua-Poly/Mount.

**Table 2:** Primary antibodies. IgG2a, immunoglobulin G2a.

**Antigen**	**Species**	**Isotype**	**Dilution**	**Source**	**Reference**
Gs	Mouse	IgG2a	1:1000	Merck	MAB302
Sox2	Mouse	IgG1	1:200	Abcam	ab171380
DsRed	Rabbit		1:250	Takara	632496
BrdU	Rat	IgG2a	1:150	Abcam	ab6326
Pcna	Mouse	IgG2a	1:500	Santa Cruz Biotechnology	PC10
GFP	Chicken	IgY	1:1000	Aves Labs	GFP-1020
Estrogen receptor α	Rabbit	IgG	1:200	Abcam	ab16660
mCherry	Chicken		1:1000	EnCor Biotechnology	CPCA-mCherry
Cleaved caspase-3	Rabbit		1:500	Cell Signaling Technology	9661

**Table 3:** Secondary antibodies.

**Antigen**	**Species**	**Isotype**	**Conjugate**	**Dilution**	**Source**	**Reference**
Chicken IgY (H + L)	Goat	IgG	Alexa Fluor 488	1:1000	Invitrogen	A-11039
Rabbit IgG (H + L)	Goat	IgG	Alexa Fluor 546	1:1000	Invitrogen	A-11010
Mouse IgG2a	Goat	IgG	Alexa Fluor 633	1:1000	Invitrogen	A-21136
Rabbit IgG (H + L)	Goat	IgG	Alexa Fluor 405	1:1000	Invitrogen	A-31556
Mouse IgG1	Goat	IgG	Alexa Fluor 647	1:1000	Invitrogen	A-21240
Rat IgG (H + L)	Goat	IgG	Alexa Fluor 488	1:1000	Invitrogen	A-11006
Mouse IgG2a	Goat	IgG	DyLight 405	1:1000	BioLegend	409209
Mouse IgG2a	Goat	IgG	Alexa Fluor 488	1:1000	Invitrogen	A-21131
Rabbit IgG (H + L)	Goat	IgG	Alexa Fluor 488	1:1000	Invitrogen	A-11008

### Image acquisition

Images of both whole-mounted telencephala and sections were acquired on confocal microscopes (LSM 700 and LSM 710, Zeiss) using either a 20× air objective or a 40× oil objective (for the comparison of *gfap*:nlsGFP and *her4.1*:dRFP expression). With the 20× air objective, we used a *z*-step of 1 μm, a numerical aperture in each channel resulting in 1.5-μm-thick optical sections, and a pixel dwell time of either 1.58 or 3.15 μs. Each line was averaged twice. With the 40× oil objective, we acquired 1-μm-thick optical sections, with a step of 0.75 μm and a dwell pixel time of 1.58 μs; no averaging was performed. The power of the lasers and the voltage of the photomultipliers (gain) were set for every single acquisition and adjusted for signal loss throughout tissue depth. For this purpose, lasers and gain were tuned at two to three different optical sections within a *z*-stack, and their values were spline interpolated and extrapolated to cover the whole thickness of the stack. Images were saved in either LSM or CZI format.

The live imaging of *gfap*:dTomato-expressing NSCs was performed on a customized commercial two-photon microscope (TriM Scope II, LaVision BioTec) equipped with an ultrafast oscillator (λ = 690 to 1300 nm; InSight DS+ from Spectra-Physics Newport). dTomato was excited with photons of 1120 nm. The fluorescent signal was collected in the backward direction and separated from the excitation wavelength by a dichroic mirror (T695lpxr, Chroma) and an interference short-pass filter (ET700SP, Chroma). Photons below and above 561 nm were separated by a dichroic mirror (Di02-R561, Semrock) and directed to the “red” detection channel equipped with a GaAsP detector (H7422-40, Hamamatsu). Before collection, the red fluorescence was further filtered using a band-pass interference filter (FF01-607/70, Semrock). To image the entire volume of interest, spanning typically 800 μm by 800 μm by 250 μm (i.e., a single brain hemisphere), we recorded mosaics consisting of four *z*-stacks arranged in a square pattern with an overlap of 10%. For each *z*-stack, the lateral field of view was 405 μm by 405 μm, the depth of imaging varied from 250 to 290 μm (starting about 250 μm below the skin), the voxel size was 0.8 μm by 0.8 μm by 2 μm, and the pixel dwell time was 4.9 μs.

### Image processing and cell counting

Images were exported to the Imaris software (versions 8 and 9; Bitplane) and converted to the Imaris file format.

#### Lineage tracing

To circumscribe the automatic detection of cells to the dorsomedial part (Dm) of the pallium (with the exception of its most posterior part), we manually drew surfaces from the most dorsal part to the most ventral part of the pallium stack, outlining the sulcus ypsiloniformis on the lateral side of Dm. The posterior limit of these surfaces was defined by the plane perpendicular to the anterior-posterior axis of the telencephalon and tangent to the posterior tips of the sulcus ypsiloniformis. The compilation of these surfaces delineated a volume, representing our region of interest (ROI), that we used to create a new specific set of channels (for every original channel, we set the voxels outside the ROI to 0). A few neurons within the parenchyma also express Sox2 and might be unduly assigned a progenitor identity (fig. S3, C and D) (*45*). To circumvent this issue, we created a new volume corresponding to the parenchyma of our ROI from which we deleted the Sox2 channel (by setting every Sox2 voxels to 0). Overall, this strategy allowed us to use the spot function of Imaris to automatically detect both mCherry- and Sox2-expressing cells. More generally, this function allows the manual or automatic registration of objects (such as cells) as “spots,” whose coordinates can be easily recovered.

In the clonal analysis experiment, we first ran an automatic detection (“segmentation”) of mCherry-expressing cells. Then, to distinguish between Sox2^+^ and Sox2^−^ cells among the mCherry^+^ population, we used a filter based on the median intensity of the Sox2 signal contained in 3-μm-diameter spheres centered on mCherry^+^ cells (spots). A similar automated approach was not feasible for the detection of Gs-expressing cells owing to the particular cytoplasmic distribution of this enzyme. As a consequence, Gs^+^/Sox2^+^ NSCs were manually distinguished from Gs^−^/Sox2^+^ NPs both on three-dimensional (3D) reconstruction of *z*-stacks and on digital cross sections, and each cell was registered with a spot corresponding to their respective progenitor category. Proliferating NSCs (aNSCs) and NPs (aNPs) were subsequently selected by applying a median intensity filter on the BrdU signal included in a 3-μm-diameter sphere around their corresponding spots. Given the complete absence of parenchymal astrocytes in the zebrafish telencephalon, the production of oligodendrocytes through an independent lineage (*46*), and the almost complete labeling of parenchymal cells by the neuronal marker HuC/D (fig. S3, A and B), all the other cells of the lineage were deemed neurons. Last, the whole population of Sox2-expressing progenitors was automatically segmented and their NNDs were calculated with the “spots to spots closest distance” function of Imaris. Both the positions and the NNDs of Sox2^+^ progenitors and the positions and identities of the traced cells were exported as Excel files for further processing. The number of Sox2^+^ cells within and outside the lineage of maximally recombined *her4.1*-expressing NSCs was determined first by automatically segmenting the whole population of Sox2^+^ cells and then by applying a filter on the basis of the median intensity of the mCherry signal within 3-μm-diameter spheres centered on Sox2^+^ spots.

#### Analysis of NSC/progenitor markers

The analysis of the distribution of the different types of pallial progenitors was carried out within a 200-μm-side cubic region included into our ROI. As Sox2 expression characterizes every progenitor contacting the pallial ventricular zone, we started by automatically segmenting Sox2-expressing cells and then sequentially applied distinct median intensity filters to the signals of the different markers until sorting out all the different progenitor categories.

In all experiments, the automatic cell segmentation was systematically verified on 3D reconstruction of the pallial *z*-stacks, and corrections were manually made whenever necessary. Furthermore, colocalizations of markers were also confirmed or edited for every single cell on digital cross sections. The telencephalic transverse sections shown in fig. S3 are displayed as single confocal planes.

#### Live imaging of NSC fates

Images recovered from live fish were analyzed as previously described (*21*). Briefly, the *z*-stacks acquired on successive imaging sessions were first converted into a single file using Imaris (Bitplane) and Fiji. Time points were then manually aligned, and intensities were adjusted to correct for potential fluctuations between imaging sessions. Between 300 and 400 cells were tracked over eight time points (23 days) in the pallial Dm region. While the approach implemented here does not allow distinguishing between loss of *gfap* expression and the death of *gfap*-expressing cells, we emphasize that apoptotic cell death was previously shown to be very low during neurogenesis in the adult zebrafish pallium (*20*).

### Statistical analysis

To ensure the consistency of the cell quantifications, the same experimenter carried out all the cell counts for a given experiment (lineage tracings, time-lapse live imaging, and analysis of NSC markers…). In addition, when comparisons were done between experiments, we also made sure that the same experimenter performed all the measurements in the experiments being compared. Investigators were not blind to the time of chase nor were they to the age of fish. No computational randomization methods were used, but special attention was paid to maximize the random distribution of fish across conditions. Balanced ratios of females and males were included in the different experimental groups as much as possible. In the clonal analysis experiments, all the fish batches (except the ones used for the 6-dpi time point) arose from crosses between the same pairs; whenever feasible, fish of induced batches were collected over several time points. In the full induction lineage tracing experiment, we used a single batch of fish that were all induced together.

Data are presented as means ± SEM, as means ± SD, as means ± 95% CI, or with box-and-whisker plots. Means represent averages per hemisphere per animal (the statistics of both hemispheres were averaged in such a way that we were able to include the brains with only one hemisphere left in the analysis). Statistical analyses were carried out using InVivoStat (*47*), and plots were created using either Microsoft Excel or R. The normality of the residuals of the responses was assessed using normality probability plots, and the homogeneity of the variance was inspected on a predicted versus residual plot (*48*). When the responses deviated noticeably from either criterion, they were first log_10_-transformed. In addition, all proportion responses were transformed using the arcsine function (*48*). Data displaying an approximately Gaussian distribution of residuals and homoscedastic responses with or without transformation were analyzed using parametric tests. When factors (e.g., time of chase and cell types) were analyzed at more than two levels (6 dpi, 18 dpi, 30 dpi…; *her4.1^+^* NSCs, *Gfap^+^* NSCs, NPs…), overall effects were determined either by analysis of variance (ANOVA) or with a repeated measures mixed model approach if animals were measured repeatedly. In this case, the within-subject correlations were modeled by a compound symmetric covariance structure. No gateway ANOVA approach was used, and pairwise comparisons were carried out independently of the results of the ANOVA with least significant difference tests. *P* values were adjusted for multiple comparisons according to the Holm’s procedure. Single comparisons were analyzed with independent (unpaired) Student’s *t* tests when the variances of the factor levels were similar, with Welch’s *t* tests when the corresponding variances were unequal, and with a paired *t* test when the two levels of the investigated factor were measured in the same animals. Responses that continued to harbor a significant deviation of their residuals from a normal distribution and/or heterogeneous variances after transformation were analyzed using nonparametric tests. In that case, overall effects were assessed with a Kruskal-Wallis test, and all pairwise comparisons were assessed with Behrens-Fisher tests (*48*, *49*). All the statistical tests performed were two-tailed, and their significance level was set at 5% (α = 0.05).

## Supplementary Material

aaz5424_SM.pdf

aaz5424_Data_file_S1.xlsx

## SUPPLEMENTARY MATERIALS

Supplementary material for this article is available at http://advances.sciencemag.org/cgi/content/full/6/18/eaaz5424/DC1
