# Supplementary material for: Lineage hierarchies and stochasticity ensure the long-term maintenance of adult neural stem cells
Source: Sci Adv. 2020 Apr 29;6(18):eaaz5424. doi: 10.1126/sciadv.aaz5424 (PMC7190328; doi:10.1126/sciadv.aaz5424)
Supplement: aaz5424_SM.pdf [file aaz5424_SM.pdf]

Science Advances
AAAS

advances.sciencemag.org/cgi/content/full/6/18/eaaz5424/DC1

Supplementary Materials for

Lineage hierarchies and stochasticity ensure the long-term maintenance of adult neural stem cells

Emmanuel Than-Trong, Bahareh Kiani, Nicolas Dray, Sara Ortica, Benjamin Simons, Steffen Rulands, Alessandro Alunni*, Laure Bally-Cuif*

*Corresponding author. Email: laure.bally-cuif@pasteur.fr (L.B.-C.); alessandro.alunni@pasteur.fr (A.A.)

Published 29 April 2020, *Sci. Adv.* **6**, eaaz5424 (2020)
DOI: 10.1126/sciadv.aaz5424

The PDF file includes:

Supplementary Materials and Methods
Supplementary Theory
Figs. S1 to S10

Other Supplementary Material for this manuscript includes the following:

(available at advances.sciencemag.org/cgi/content/full/6/18/eaaz5424/DC1)

Data file S1

Supplementary Materials

Supplementary materials and methods

Brain region analyzed

The adult zebrafish telencephalon harbors a large neurogenic domain including the territories thought to be homologous to the two NSC-hosting niches of the mammalian brain (the subependymal zone of the lateral ventricle and the subgranular zone of the dendate gyrus). We decided to focus our study on the dorsal part of the pallial Dm region (Fig. 1A, S1A and S1C). This region harbors numerous dorsally exposed NSCs, easily accessible for whole-mount immunohistochemistry and subsequent confocal imaging, as well as for intra-vital imaging. Furthermore, in Dm, the minimal migration of their neuronal progeny allows clones to remain compact and close to the surface which, beyond alleviating potential clonal ambiguities, also permits to capture the whole set of clones in a single acquisition without the need to resort to sectioning or tissue clearing. Finally, Dm is currently the best characterized pallial germinative area in zebrafish at the molecular level and has the advantage of harboring NSCs with a greater activation frequency (*21*), thus enhancing our chances to capture the clones' dynamics in this overall relatively slow proliferating system (*19, 28, 50*). Relative to mammalian neuroanatomical subdivisions, Dm encompasses the neocortical area, which is neurogenic in adult zebrafish (*23*).

Clonality of the last time point

The last time point of the clonal analysis (507 dpi) displayed a significant increase of its average number of clones relative to previous time points, thus raising concerns about its clonality (Fig. S4E). Indeed, this increase might reveal an under-clustering of the cells caused by a possible fragmentation of the clones. In fact, both the division of unlabeled progenitors and the generation of new unmarked neurons in the close vicinity of the traced clones can contribute to the scattering of their comprising cells. However, we argue that the main conclusions that we drew about the clonal dynamics at 307 dpi – i.e. the appearance of a plateau both in the number of NSCs per NSC-containing clone and in the proportion of NSC-containing clones – still hold at 507 dpi. Notably, as 507 dpi clones are mainly comprised of neurons, their fragmentation would be expected to lead to a decrease in the proportion of NSC-containing clones, which was not the case. Furthermore, while the expanded neuronal content of the clones at 507 dpi might have blurred the unambiguous assignment of their cells, their NSC were scored with high confidence owing to the lower fraction of NSC-containing clones at that time. Hence, based both on our high confidence in the maintenance of the plateau in the number of NSCs per NSC-containing clones and on the stability of the proportion of NSC-containing clones - which contradict a fragmentation of the clones-, we concluded that the clone dynamics observed at 507 dpi were reliable. They were thus included in our analysis.

Homeostasis of NSCs within the her4 lineage

The results presented in figure 2E and 2F suggest that NSCs within the *her4:ERT2CreERT*-traced lineage are in homeostasis. However, the relatively important inter-individual variation in the number of induced clones may have blurred significant changes in the number of traced NSCs with advancing time of chase. Accordingly, it is indeed surprising that the proportion of NSC-containing clones dropped by a factor of 5 between the beginning of the chase and the time when the plateau was reached, whereas the number of NSCs within NSC-containing clones only doubled (Fig. 3C-D and S6A-B). Notably, from the apparent

homeostasis of the traced NSC population, one would have expected a complete compensation of NSC losses (i.e. that the number of NSCs per NSC-containing clone plateaus at 5 instead of 2). In addition, the difference between the proportions of rNSCs/oNSCs (61%/39%) predicted by the model and the ones inferred from the experimental data (20%/80%), suggests the existence of a bias of the induction process toward the oNSC population (see also Fig. S2). Because the fates of oNSCs appear overall tilted toward neuronal differentiation, such a bias would also be expected to result in an initial reduction in the number of traced NSCs (i.e. at early chase times, before the dynamics becomes dominated by rNSCs and their oNSC progeny). We thus considered the possibility that inter-individual variability in induction frequency (i.e. in clone numbers) together with the low number of traced NSCs under clonal conditions (mean: 15; s.d.: 4.3) would have masked an initial reduction in the number of NSCs within the *her4* lineage. To account for the effect of clone number on the amount of traced NSCs, we normalized the number of traced NSCs by the number of clones. This revealed a significant decrease in the average number of NSCs per clone at early time points, which was then followed by a plateau around 0.45 NSCs/clone (Fig. S10). This result does not contradict in any way the conclusion that *her4*$^+$ NSCs are intrinsically homeostatic. Indeed, the appearance of a plateau in the average number of NSCs per clone after a transitory period of decline confirms both the heterogeneous nature of *her4*-expressing NSCs as well as the long-term self-renewal potential of part of them. Further, the bulk lineage tracing of *her4*-expressing NSCs – which holds the double advantage of minimizing the variability in the number of targeted NSCs and of being unbiased (as most *her4*$^+$ NSCs are marked) – confirmed the homeostatic nature of their lineage (Fig. S5C-E). Finally, it is also interesting to note that the proportions of rNSCs/oNSCs predicted by the model are expected, assuming homeostasis of NSCs within the *her4* lineage, to result in a plateau at about 1.64 NSCs per NSC-containing clones. It is remarkable that this value, which, within the frame of the proposed hierarchy, is independent of any induction bias, converges toward the experimentally observed value (Fig. 3C and S6A).

Hierarchical organization of rNSCs and oNSCs

Beyond the ability of our minimal model to quantitatively account for the observed clonal dynamics, the hierarchical relationship between rNSCs and oNSCs is also supported by a number of additional arguments:

- the maintenance of a plateau in the number of NSCs per NSC-containing clones. Indeed, whereas the existence of two parallel lineages of rNSCs and oNSCs would also result in a plateau in the fraction of NSC-containing clones, only their hierarchical organization can lead to plateauing for the number of NSCs per NSC-containing clones. Notably, in the absence of major changes in NSC quiescence and fates during the time frame of the experiment (Fig. S6C and S8D), the unbalanced fates of oNSCs would, after a transient increase in the number of NSCs per NSC-containing clones (a consequence of their population dynamics), lead to a collapse of this parameter as oNSCs would progressively exhaust. On the long-term, clones retaining NSCs would be exclusively comprised of rNSCs and their NSC content would tend towards 1 (as rNSCs divide asymmetrically). Finally, at the level of the entire *her4* lineage, the disconnection between the rNSC and the oNSC lineages would result in a substantial shrinkage of the *her4*-derived NSC population, again owing to the unbalanced fate of oNSCs. This outcome is also ruled out by the bulk lineage tracing of *her4*$^+$ NSCs, which demonstrate their homeostatic behavior (Fig. S5C-E);

- the generation of neurons at a constant rate relative to the number of traced NSCs. Indeed, should the oNSC and rNSC pools not be hierarchically organized, one would expect the neuron/NSC ratio to progressively change as oNSCs become exhausted.

Supplementary Theory

Here we give further details on the calculations supporting the main conclusions drawn in the main text.

Assignment of clonal origin to cells

The interpretation of lineage tracing data in the zebrafish pallium is complicated by the fact that stochastic forces originating from cell divisions in the surrounding tissue can lead to clone fragmentation and dispersion, which ultimately leads generic scaling distributions of clone sizes and an erasure of biological information (*31*). As neither the number of induced cells nor the rate of fragmentation are known a priori this renders the assignment of clonal progeny potentially ambiguous. By implementing a mathematical framework to analyze the spatial statistics of dispersed cells it is possible to recover the clonal origin of marked cells with known uncertainty and to unveil information on cell fate behavior of the traced population. We emphasize that this framework provides a general platform to assign the clonal provenance of marked cells in parallel contexts.

If induction events are statistically independent, the number of induced cells, n, in a given pallial hemisphere is distributed according to a Poisson distribution,

$$P(n;\lambda) = \frac{\lambda^n}{n!} e^{-\lambda},$$

where λ denotes the induction frequency. Then, in two spatial dimensions, the distance d to the nearest neighbor of an induced cell is distributed according to

$$g(d;\lambda) = \frac{d}{\lambda^2} e^{-\frac{d^2}{2\lambda^2}}.$$

As cells divide and clones expand and disperse, the positions of labelled cells cease to be statistically independent such that the distances between nearest neighbors of labelled cells are not distributed according to $g(d;\lambda)$ for any value of λ. However, if stochastic forces from the surrounding tissue are isotropic, the centers of marked clones defined by the average position of cells, $(\bar{x},\bar{y}) = N^{-1}\sum_i (x_i, y_i)$, remain statistically independent and nearest neighbor distances are distributed according to $g(d;\lambda)$. Therefore, for a given putative clonal assignment of cells the agreement of the distribution of nearest neighbors of clone centers with $g(d;\lambda)$ can serve as a test for the correctness of this assignment, such that the true clonal assignment has the highest accordance with the assumption of statistical independence of clone centers.

To formalize this basic idea, we begin by considering the probability that a given partition of a set of cells into clones, Π, is clonal. Using Bayes theorem, we can write this probability as the product of the probability of observing the experimental data (the coordinates of labelled cells) given the clonal assignments encoded in a partition, Π, times our prior believe in a clonal partition,

$$P_1(\Pi|\{x_i, y_i\}) = \frac{L(\{x_i, y_i\}|\Pi)G(\Pi)}{\int L(\{x_i, y_i\}|\Pi)G(\Pi)\mathrm{d}\Pi}.$$

The denominator ensures normalization. The likelihood function, $L(\{x_i, y_i\}|\Pi)$, contains the data-dependent part of the denominator, while the prior, $G(\Pi)$, encodes our a priori belief that a given partition is clonal. If induction events and cell fates in distinct clones are each statistically independent, the likelihood function takes the form

$$L(\{x_i, y_i\}|\Pi) = \prod_{i=1}^{n} g(d_i; \lambda),$$

where the product goes over all putative clones in the partition Π and d_i are the nearest neighbor distances of their respective centers of mass. The parameter λ is determined self-consistently from the number n of putative clones in the partition by $\lambda = 1/\sqrt{2\pi\rho}$, with ρ being the number of putative clones in the partition Π divided by the area of the analyzed tissue sample. Intuitively, the likelihood is therefore a measure for the accordance of the clonal partition Π with the hypothesis of independent labelling.

The assignment of the clonal provenance of labelled cells can be improved by considering further knowledge we might have on cell proliferation or cell migration. Specifically, in the pallium, there are biological and physical limits on cell migration, and therefore on the degree of clone dispersion. Cells are subject to stochastic forces exerted by the surrounding tissue leading to their diffusive displacement (*51*). If D is the corresponding effective diffusion constant, cell positions at a time t after labelling are approximately normally distributed,

$$P(r, t) \approx \frac{1}{\sqrt{8\pi Dt}} e^{-\frac{\mathrm{r}^2}{8Dt}},$$

where r is the distance from the initial position of the induced cells. With this, we consider a tissue sample containing n putative clones. Then, the maximum distance to the clone centre across all clones in the tissue is obtained by calculating the n-th order statistics, i.e.

$$G(\max r, t) = \frac{2^{\frac{1}{2}-n}}{\sqrt{\pi 4Dt}}\, n\, e^{-\frac{(\max r)^2}{8Dt}} \left(1 + \frac{2}{\sqrt{\pi}} \int_0^{-\frac{\max r}{\sqrt{8Dt}}} e^{-y^2}\mathrm{d}y\right)^{n-1}.$$

For the tail of the distribution, $\max r \gg \sqrt{Dt}$, this expression is well approximated by

$$G(max\, r, t) \approx \frac{1}{\sqrt{8\pi Dt}} e^{-\frac{(max\, d_i)^2}{8Dt}} \equiv G(\Pi),$$

which we used as a prior in $P_1(\Pi|\{x_i, y_i\})$. We calibrated the effective diffusion constant, D, by selecting a sample where clonal assignments of labelled cells could be unambiguously made by visual inspection. We then determined D such that the number of clones counted by visual inspection was equal to the algorithmically calculated number of clones (see below) and obtained $D = 1.8\, \mu m^2/d$. This means that, on average, within 100 days a labelled cell covers an area of 180 μm^2 as a result of stochastic forces exerted by the surrounding tissue.

As the posterior probability trivially depends on the number of terms comprising the log likelihood, and therefore on the number of assigned clones in the partition, Π, we compare P_1 to the posterior probability P_0 in a scenario, where the hypothesis of statistical independence of clone centers is true. We therefore seek the clonal partition that maximize their ratio,

$$K = \frac{P_1(\Pi|\{x_i, y_i\})}{P_0(\Pi|\{x_i, y_i\})}$$

In this case, we can calculate the posterior analytically,

$$P_0(\Pi|\{\mathrm{x_i}, \mathrm{y_i}\}) = \frac{1}{\sqrt{\pi}8Dt}\left(\frac{\sqrt{\pi}}{4\lambda}\right)^n.$$

As the number of possible partitions of labelled cells into clones is too large to be computationally accessible, we employ a hierarchical or coarse-graining approach. Starting from a partition, where the number of clones is equal to the number of labelled cells, at each iteration we merge the two clones whose centers of masses have the smallest distance. While this approach bears the risk that the true clonal partition might not be tested, it allows us to obtain an approximation of this partition within only n iterations. The partition with the maximum value of K is taken for downstream analysis. To estimate the uncertainty associated with the assignment of clonality, we define all partitions whose value of K exceeds a threshold,

$$\Pi_{clonal} = \left\{\Pi|\log K > \max_{\Pi} \log(K) - \log\alpha\right\}$$

as credibly clonal.

We set α to 0.05 such that partitions having a value of K exceeding 5% of the maximum value are considered credible. We found that for all analyzed samples the mode of the posterior distribution and the corresponding credibility bounds were well defined. For each pallial hemisphere analyzed, subsequent clonal analysis was performed for the partition corresponding to the maximum value of K and for those corresponding to the lower and upper boundaries of the credibility interval.

Modelling of the clonal dynamics

<u>Maximum likelihood estimation</u>

To unveil the dynamical rules that underlie NSC fate regulation in the pallium, we employ the idea of maximum likelihood estimation. Specifically, we seek to identify the model, defined by a set of parameters $\Theta = (\Theta_1, \Theta_2, \dots)$, that has the highest probability, $P(\Theta|E)$, given the experimental data, E. According to Bayes' theorem, this probability is proportional to the probability $P(E|\Theta)$ of obtaining the experimental evidence if a certain model Θ is true while $P(\Theta)$ gives prior belief in the model,

$$P(\Theta|E) = \frac{P(E|\Theta)P(\Theta)}{\int_\theta d\Theta\, P(E|\Theta)P(\Theta)}.$$

The denominator ensures normalization. The probability $P(E|\Theta)$ is called likelihood, $L_E(\Theta) = P(E|\Theta)$. Without prior knowledge, i.e. if the prior $P(\Theta)$ follows a uniform

distribution, the principle of maximum likelihood states that the model with the highest probability is the one that maximizes the likelihood function, $L_E(\Theta)$.

In our clonal labelling assay, the experimental evidence is given by the frequency $f_{n_s,n_n,t}$ of observations of clones with n_s NSCs ("s" stands for stem cells) and n_n neurons at time t post induction. Since the clonal observations are statistically independent, we can rewrite the likelihood function as

$$L_{n_s,n_n,t}(\Theta) = N^{-1} \prod_{t} \prod_{n_s,n_n} P(n_s, n_n, t|\Theta)^{f_{n_s,n_n,t}},$$

with

$$N^{-1} = \frac{\left(\sum_{n_s,n_n} f_{n_s,n_n,t}\right)!}{\left(\prod_{n_s,n_n} f_{n_s,n_n,t}\right)!},$$

where the prefactor N^{-1} is determined by normalization and the products and the sums go over all time points and all clonal compositions.

In order to obtain the likelihood function, we need to calculate the probability $P(n_s, n_n, t|\Theta)$ to find a clonal composition with n_s NSCs and n_n neurons at time t. The time evolution of this probability is given by a master equation of the form,

$$\frac{d}{dt}P(n,t|\Theta) = \sum_{n_s',n_n'=0}^{\infty} \omega_{n',n}P(n',t|\Theta) - \omega_{n,n'}P(n,t|\Theta).$$

The transition rates, $\omega_{n,n'}$, between states with different cellular composition carry the details of the model. For simple cases the master equation can be solved analytically, however, generally, numerical methods are necessary to find approximate solutions. Using Gillespie's algorithm, we generated stochastic trajectories whose accumulation provided histograms and consequently the probability distribution $P(n_s, n_n, t|\Theta)$. As extinct clones $(n = 0)$ are experimentally undetectable we define the clone size distribution of NSC-containing clones,

$$P_{n>0}^{m}(n, t|\Theta) = \frac{P(n,t|\Theta)}{1-P(0,t|\Theta)}.$$

Since maximizing the likelihood function is equivalent to maximizing its logarithm, we consider the logarithm of the likelihood function,

$$\log L_E(\Theta) = \log N^{-1} + \sum_{t} \sum_{n_s,n_n,t} N_{n_s,n_n,t} \log P_{n>0}^{m}(n_s, n_n, t|\Theta).$$

As the normalization factor N^{-1} is independent of the model parameters, we can neglect this prefactor in the subsequent analysis. Therefore, by substituting a particular set of experimental data in the probability distribution $P(n, t|\Theta)$, we obtain the value of the likelihood function for a given model.

Uncertainty and credibility intervals

The finite size of the experimental and numerical samples and the variability between fish generate uncertainty in the estimation of the model parameters. To represent this uncertainty, we present the estimated parameters with margins. We define all parameters whose likelihood is above a given threshold α as plausible, resulting in a credibility interval,

$$CR = \left\{ \Theta \middle| \frac{L_E(\Theta)}{L_E(\Theta^*)} > \alpha \right\}.$$

Here, we chose $\alpha = 0.05$, meaning that parameter values, whose likelihood is more than 5% of the value of the maximum likelihood, are considered credible. Following this, we present the value of each parameter Θ_i with its credibility interval, $\Theta_i^* \pm (max_{CR}\{\Theta_i\} - \Theta_i^*, \Theta_i^* - min_{CR}\{\Theta_i\})$.

Inference of the best fitting model

NSC compartment

To begin, we focused our analysis on NSCs ($Sox2^+$ cells) alone. Motivated by the observed plateau in the fraction of persisting NSC-containing clones, we hypothesized that the NSC population is heterogeneous and contains a subpopulation of long-lived reservoir NSCs (hereafter denoted as A* cells), giving rise to a downstream population of operational NSCs (referred to as A cells), whose fates are overall biased towards differentiation. In order to give rise to a plateau in the fraction of surviving clones, labelled A^* need to be maintained over the time of the experiment in such a way that loss via a potential neutral drift is negligible. The saturation in the average sizes of NSC-containing clones (Fig. 3C) suggests that the A* population is not downstream of A (in which case NSC-containing clones would accumulate NSCs over time) but feeds into the A population. The simplest way the A* population can be maintained over long times while at the same time giving rise to cells of type A is via asymmetric cell divisions. We denote the rate with which A^* divides asymmetrically to give rise to both another A^* and an A cell by ν. We note that while an asymmetrically dividing populations of reservoir NSCs is the simplest process in agreement with the clonal data, we cannot rule out a more complex cell fate behavior in this compartment, such as the existence of a closed niche or further heterogeneity.

The size of the A compartment can only increase by symmetric proliferating divisions or decrease due to symmetric differentiating divisions or direct differentiations. We denote the rates for both processes with λ and μ, respectively. If these operational NSCs reverted back to the reservoir NSC compartment to maintain homeostasis such a process would necessarily need to be balanced by a loss of reservoir NSCs, for example by direct differentiation to operational NSCs. Such a process would, however, lead to clonal loss due to chance fluctuations and is therefore incompatible with the clonal data. Reversion of operational NSCs therefore must be rare or nonexistent. In summary, the dynamical rules governing cell fates of the traced NSC population can be written in chemical notation as:

$$A^* \longrightarrow A^* + A \qquad \text{With rate } \nu,$$

$$A \longrightarrow A + A \qquad \text{With rate } \lambda,$$

$$A \longrightarrow \emptyset \qquad \text{With rate } \mu.$$

Performing maximum likelihood estimation, we obtain the best fit for parameters corresponding to a log likelihood of $\log L_E(\Theta^*) = -791.55$, with rates

$$\nu = 0.007 \pm (0.005{,}0.0007\ )\ \mathrm{d}^{-1},$$

$$\lambda = 0.006 \pm (0.003, 0.001)\ \mathrm{d}^{-1},$$

$$\mu = 0.017 \pm (0.012, 0.003)\ \mathrm{d}^{-1}.$$

In summary, we find the best fitting parameters defining a model for a heterogeneous population of NSCs consisting of two subpopulations where, on average, A^*cells give rise to A cells every $143 \pm (15.87{,}59.52)$ days, A cells duplicate every $166.67 \pm (33.30, 55.56)$ days and are lost through neuronal differentiation every $58.82 \pm (12.6, 24.34)$ days. In the field of stochastic processes, this model is often referred to as a birth-death process with immigration(*52*). If $\lambda < \mu$, as is the case here, this model ultimately gives rise to a steady state in the clone size distribution, which, in the long-term, is representative of the tissue. The best fitting parameters suggest that the traced NSC population is overall homeostatic, an observation that we were able to independently confirm by our experiments (Fig. 2E-F and 5E). Thus, in the steady state the ratio of A* to A cells is $= (\mu - \lambda)/\nu = 1.57 \pm (0.095{,}0.3)$, such that the traced NSC population is comprised for 61% of A* cells (reservoir NSCs) and for 39% of A cells (operational NSCs).

To challenge the hypothesis of heterogeneity in the NSC population, we asked whether alternative models involving a homogeneous (equipotent) NSC population could equally explain the clonal data. First, such a model would have to predict the stochastic dynamics of NSC-containing clones. The quality of such a prediction is measured by the value of the maximum of the log likelihood. In order to quantify the relative capacity of different models to describe the clonal data we estimated the relative information lost in describing the experimental data in each case, a quantity known as Akaike information criterion (AIC). Secondly, in addition to the statistics of NSC-containing clones, these models need to predict the time evolution of lost clones, in particular the plateauing of the fraction of surviving clones.

In the simplest case, if the NSC population is equipotent the dynamics is determined by a simple birth-death model similar to the one proposed in other systems(*33*). In chemical notation,

$$A \longrightarrow A + A \qquad \text{with rate } \lambda$$

$$A \longrightarrow \emptyset \qquad \text{with rate } \mu.$$

The maximum of the log likelihood is -793.2. Calculating the Akaike Information Criterion (AIC) for each of the two models (indices 1 and 2 respectively refer to the heterogeneous and the homogeneous NSC populations), we obtain $AIC_1 = 1589$ and $AIC_2 = 1590$. This means that, after considering the different number of parameters estimated in each case, the equipotent model is by a factor of 0.6 less likely to minimize information loss in describing the clonal data, in favor of NSC heterogeneity. While the maximum values of the log likelihoods are relatively similar, such a model containing an equipotent NSC population necessarily leads to a rapid loss of labelled clones and therefore is incompatible with the observation of a plateauing fraction of NSC-containing clones over long times.

A different scenario that can result in the observed non-zero plateau with a homogeneous NSC population is a closed-niche model based on the regulation of NSC fates by their number in the niche, as suggested by Basak et al. (*15*) for the adult mouse sub-ependymal zone. In this model, the probability that a NSC follows either proliferation or differentiation, is coupled to the number of NSCs present in the niche,

$$A \longrightarrow A + A \qquad \text{with rate } \lambda\, f(n_A, n_B, t),$$

$$A \longrightarrow \emptyset \qquad \text{with rate } \lambda\,(1 - f(n_A, n_B, t)),$$

where $f(n_A, n_B, t) = \exp[-\ (n_A + n_B - n_0)/n]$ is the probability of symmetric proliferation and n_A and n_B are, respectively, the number of labeled and unlabeled cells in the niche at time t. n_0, n and λ are parameters of the model. Our simulations demonstrate that although this model can result in a plateau for the average number of NSCs in NSC-containing clones, it can poorly resolve the value of the plateau in the fraction of NSC-containing clones and the time scale over which it is reached. The value of the maximum log likelihood for this model is $\log L_E(\Theta^*) = -817.88$, meaning that the niche-based model is 1.65×10^{-24} fold less likely than the one comprising a heterogenous population. It is important to note that this does not rule out the existence of a closed niche governing a heterogeneous NSC population as discussed above.

To account for a potentially biased cell labelling upon induction, we initialized our simulations in such a way that clone sizes and cell type compositions resembled the clonal data from brains taken shortly after labelling (at 6dpi time). We then made predictions for all subsequent time points.

Neuron compartment

Having determined the dynamical rules and parameters governing cell fate behavior of the traced NSC population, we then asked whether such a model could predict the neuronal output of the labelled NSC population over time. To this end, we considered all the possible fates by which NSCs could give rise to neurons,

$$A^* \longrightarrow A^* + A \qquad \text{With rate } \nu,$$

$$A \longrightarrow A + A \qquad \text{With rate } \lambda,$$

$$A \longrightarrow A + N \qquad \text{With rate } \kappa,$$

$$A \longrightarrow N + N \qquad \text{With rate } \mu_1,$$

$$A \longrightarrow N \qquad \text{With rate } \mu_2.$$

and used the maximum likelihood parameters obtained for the NSC model ($\nu = 0.007$ d^{-1}, $\lambda = 0.006$ d^{-1}, and $\mu_1 + \mu_2 = 0.017$ d^{-1}). We applied maximum likelihood estimation to predict the neuronal output of operational NSCs. Using the best fitting parameters, $\kappa = 0.018 \pm (0.0007, 0.0009)$ and $\mu_1 = 0.004 \pm (0.0001, 0.0003)$, and the steady state value for the ratio of A^* to A cells ($r = 1.57$), allowed us to quantify the fractions of different fate outcomes. Specifically, the probabilities of different fate outcomes are related to the rates of symmetrical, asymmetrical, and direct differentiating events,

$$P_{NSC/NSC} = \frac{(\lambda + rv)}{(\lambda + rv) + \kappa + \mu},$$

$$P_{NSC/n} = \frac{\kappa}{(\lambda + rv) + \kappa + \mu},$$

$$P_{n/n} = \frac{\mu_1}{(\lambda + rv) + \kappa + \mu},$$

$$P_n = \frac{\mu - \mu_1}{(\lambda + rv) + \kappa + \mu},$$

where $P_{NSC/NSC}$, $P_{NSC/n}$, $P_{n/n}$, and P_n are, respectively, probabilities of NSC/NSC, NSC/n, n/n, and n fate outcomes. Our analysis shows that NSC/NSC divisions constitute $P_{NSC/NSC} = 33 \pm (0.05,0.1)\%$ while NSC/n, n/n and n constitute, respectively, $P_{NSC/n} = 35 \pm (0.01,0.1)\%$, $P_{n/n} = 7 \pm (0.03,0.04)\%$ and $P_n = 25 \pm (0.05,0.01)\%$ of all possible fates. As demonstrated in Figure 4E, these percentages are in good agreement with the values obtained via our live imaging experiments.

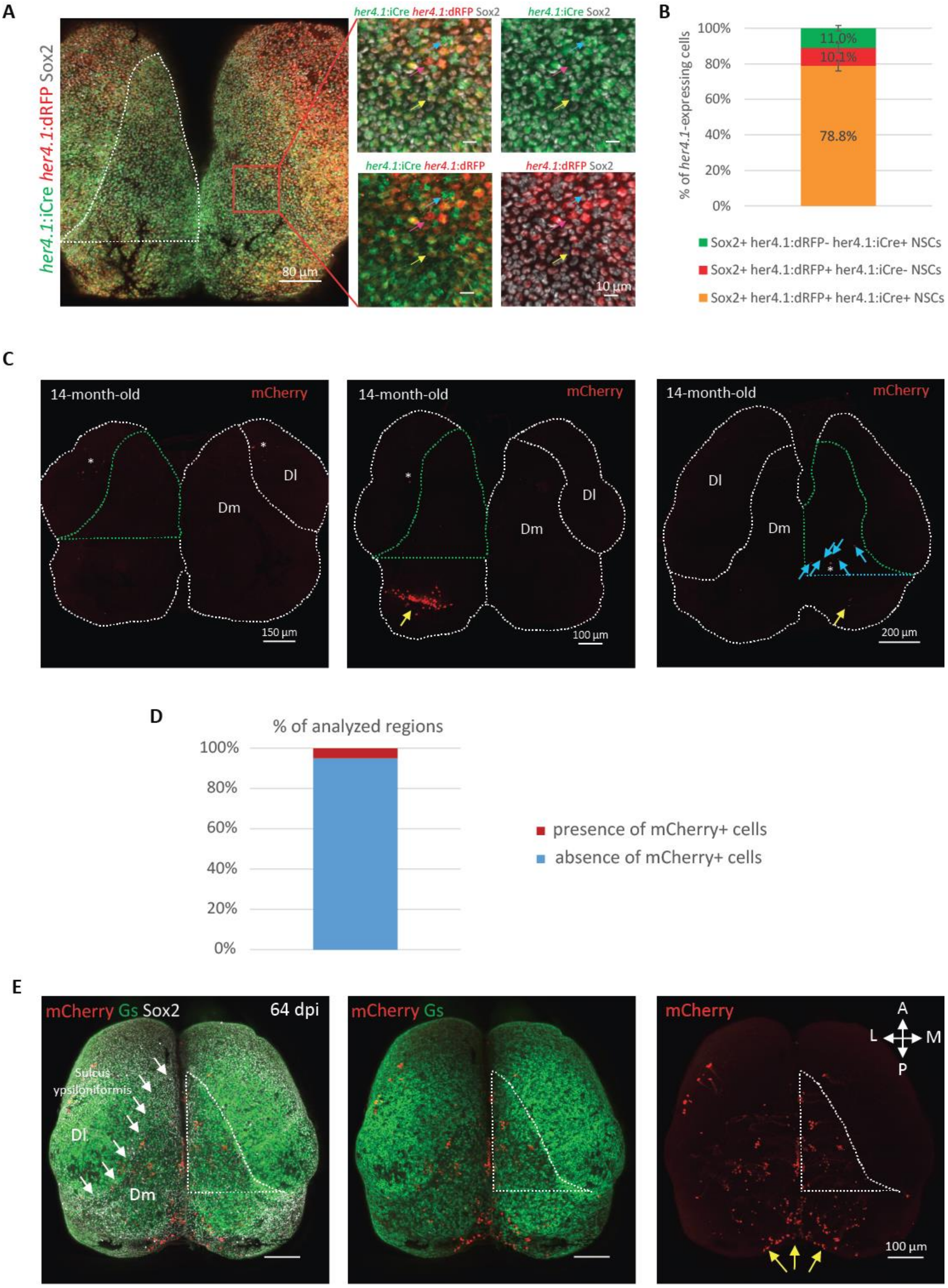

Fig. S1. Characterization of the *her4.1:ERT2CreERT2*-traced cell population. (A) Whole-mount dorsal view of the pallium of a *her4.1:ERT2CreERT2;her4.1:dRFP* double transgenic adult immunostained for ERT2, dRFP and Sox2. The Dm domain analyzed is

delineated by the dotted lines. Close-ups of the boxed area (split channels) illustrate dRFP$^+$/ERT2$^+$ (yellow arrow), dRFP$^+$/ERT2$^-$ (pink arrow) and dRFP$^-$/ERT2$^+$ (blue arrow) NSCs. (**B**) Distribution of *her4.1*:dRFP$^+$ and *her4.1:*ERT2CreERT2$^+$ among all Sox2$^+$ cells expressing *her4.1*. n=4 brains. Error bar: s.e.m. (**C**) Left: Representative dorsal view of 7 out of 10 pallia showing the complete absence of recombination of the *ubi:Switch* transgene in the entire pallial area of non-induced *her4.1:ERT2CreERT2;ubi:Switch* double transgenic zebrafish. Center: Dorsal view of an non-induced pallium harboring mCherry-labelled cells in its most posterior part (yellow arrow), indicating spontaneous recombination of the *ubi:Switch*. 3 out of 10 pallia displayed recombination outside the analyzed region. The pallium shown here is the one exhibiting the highest number of mCherry$^+$ cells. Right: Dorsal view of the unique case showing recombined cells in the region of interest (6 cells in a single hemisphere - blue arrows). The yellow arrow points to recombined cells located outside the region of interest. The pallium as well as the boundary between Dm and Dl are highlighted with white dotted lines. The region analyzed in the clonal analysis is delineate in green. * mark artifacts. Fish were analyzed at one year and two months of age so as to cover most of the time span by the clonal analysis. (**D**) Proportion of hemispheres displaying mCherry$^+$ cells in the region analyzed (n=20 hemispheres from 10 brains). (**E**) Dorsal whole-mount view of a pallium induced at 3 mpf and immunostained at 64 dpi for mCherry (red), Gs (green) and Sox2 (grey). White arrows show the sulcus ypsiloniformis (sy) that separates Dm from Dl. Yellow arrows point to several traced cells in the most posterior part the pallium whose clonal affiliation cannot be unambiguously ascertained. Such equivocal cell clusters resulted from a higher induction rate in this particular region and led us to exclude it from of our clonal analysis. The dotted area indicates the Dm territory selected for analysis. Its posterior border is identified by anatomical landmarks (posterior limit reached by the sy).

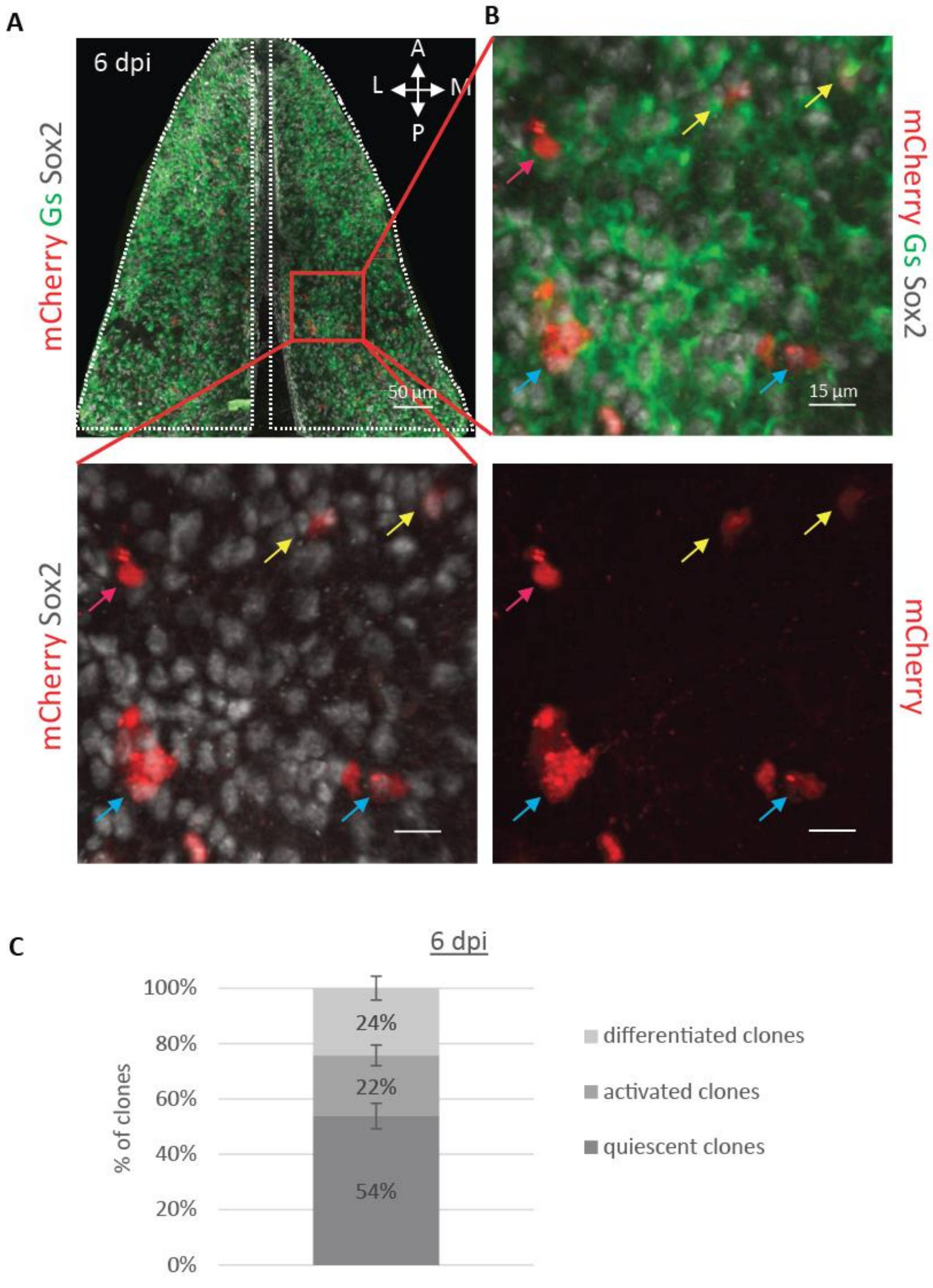

Fig. S2. Activation state of clones at 6 dpi. (**A**) Dorsal view of the pallial Dm region analyzed illustrating the different types of clones (red, mCherry^{+}) present at 6 dpi. NSCs are Gs^{+} and Sox2^{+}, NPs are Sox2^{+} only. (**B**) Higher magnification of the boxed area in (A). Blue arrows point to active clones (harboring at least two cells and comprising at least one Sox2^{+} cell), yellow arrows to quiescent clones (comprised of a single Sox2^{+} cell) and magenta arrows to differentiated clones (devoid of Sox2^{+} cell). (**C**) Distribution of clones at 6 dpi based on their activation state. n=6 brains.

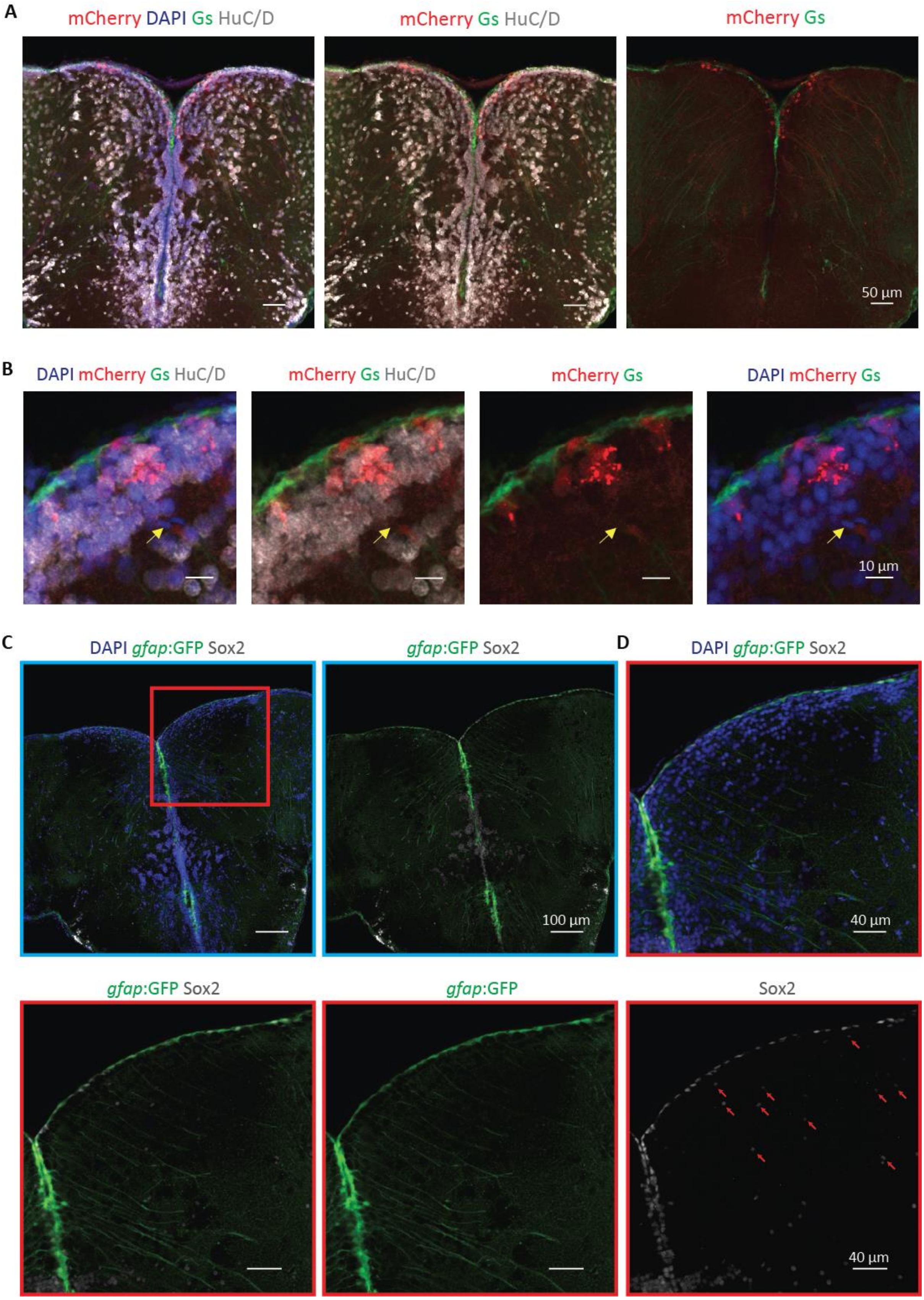

Fig. S3. Parenchymal traced cells are neurons. (**A**) Cross-section of the telencephalon of an *her4.1:iCre;ubi:Switch* fish induced at 3 months and immunostained for mCherry (red),

Gs (green) and neuronal marker HuC/D (grey). Note that almost all the parenchymal cells stain for HuC/D. (**B**) Closeup on some traced cells. The yellow arrow points to putative endothelial cells recognizable by their small flattened nuclei. (**C**) Cross-section through the telencephalon of a *gfap*:GFP transgenic fish immunostained for GFP and Sox2. Nuclei were stained with DAPI. (**D**) Closeup corresponding to the region framed in red in (C) and illustrating the presence of a few cells weakly expressing Sox2 within the pallial parenchyma. Red arrows point to some parenchymal $Sox2^+$ cells.

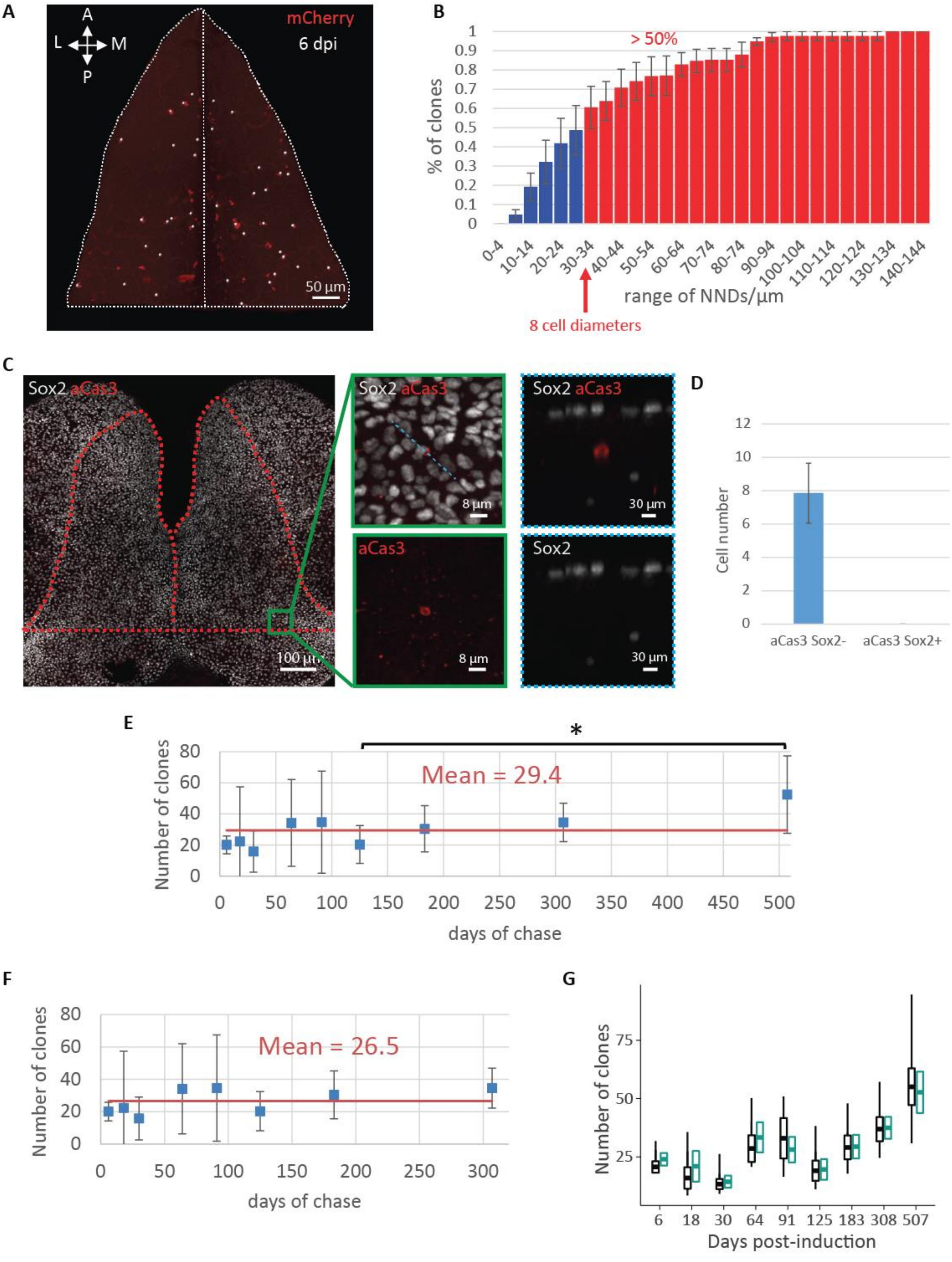
A
A
L
M
P
mCherry
6 dpi
50 µm
B
> 50%
% of clones
range of NNDs/µm
8 cell diameters
0-4
10-14
20-24
30-34
40-44
50-54
60-64
70-74
80-74
90-94
100-104
110-114
120-124
130-134
140-144
C
Sox2 aCas3
100 µm
8 µm
aCas3
30 µm
Sox2
D
Cell number
aCas3 Sox2-
aCas3 Sox2+
E
*
Mean = 29.4
Number of clones
days of chase
F
Mean = 26.5
Number of clones
days of chase
G
Number of clones
Days post-induction
6
18
30
64
91
125
183
308
507

H

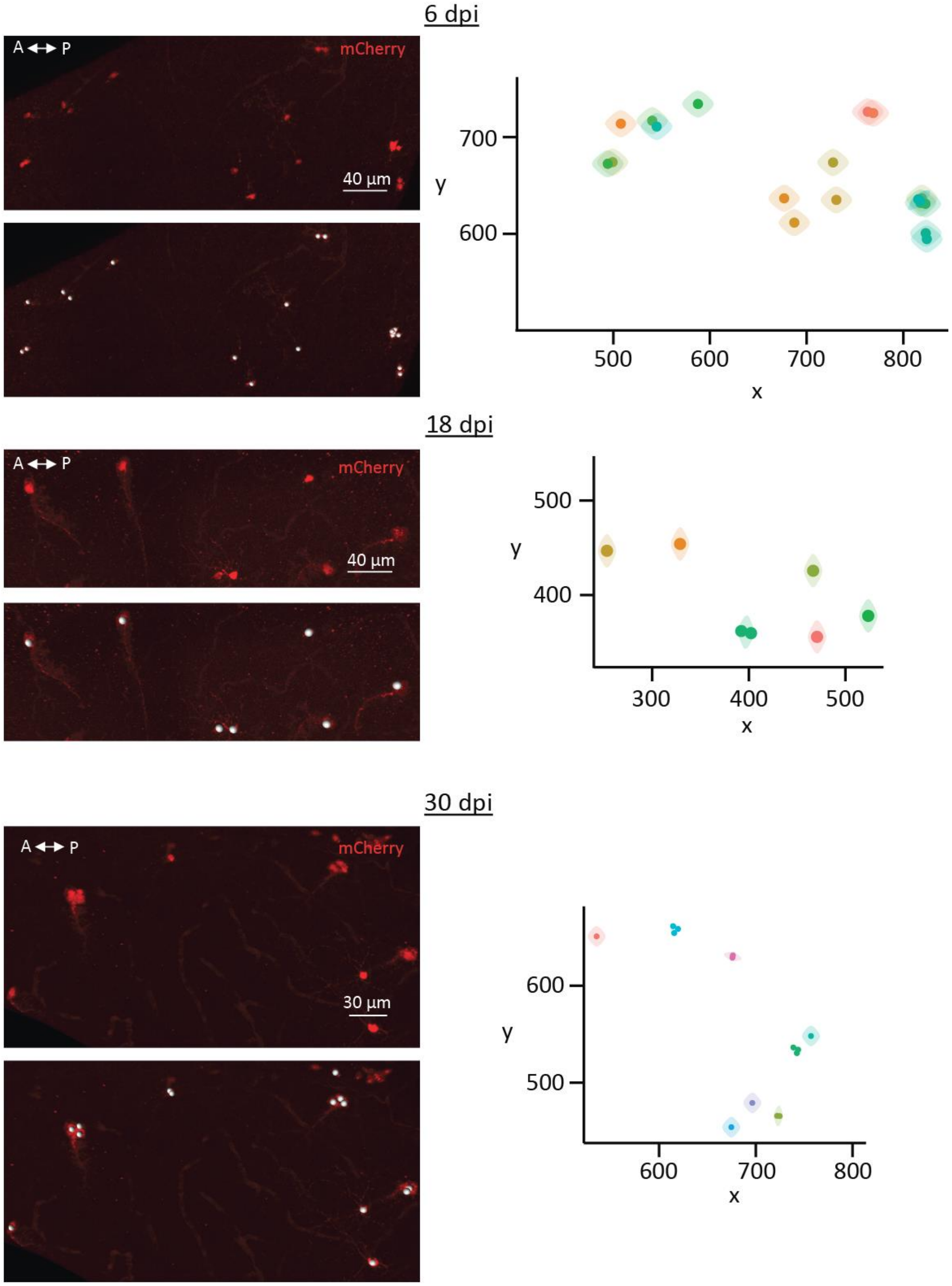
6 dpi
A ↔ P
mCherry
40 µm
700
600
y
500
600
700
800
x
18 dpi
A ↔ P
mCherry
40 µm
500
400
y
300
400
500
x
30 dpi
A ↔ P
mCherry
30 µm
600
500
y
600
700
800
x

64 dpi

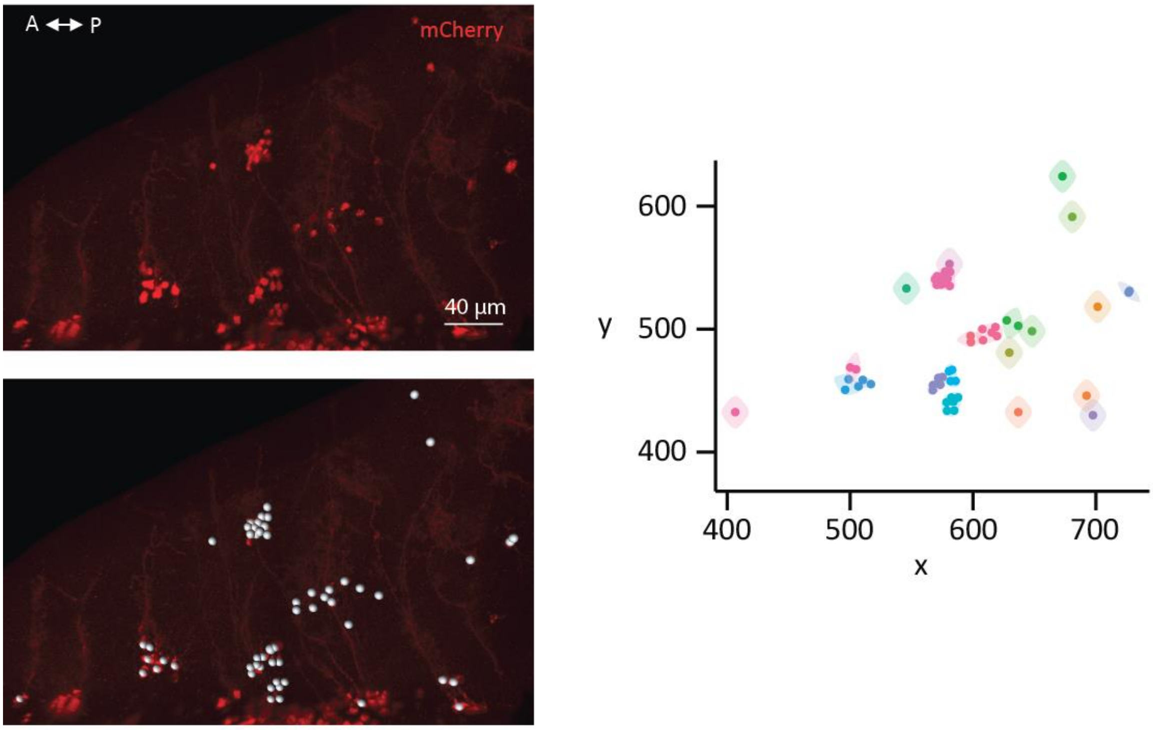
A ↔ P
mCherry
40 µm
600
500
400
y
400
500
600
700
x

91 dpi

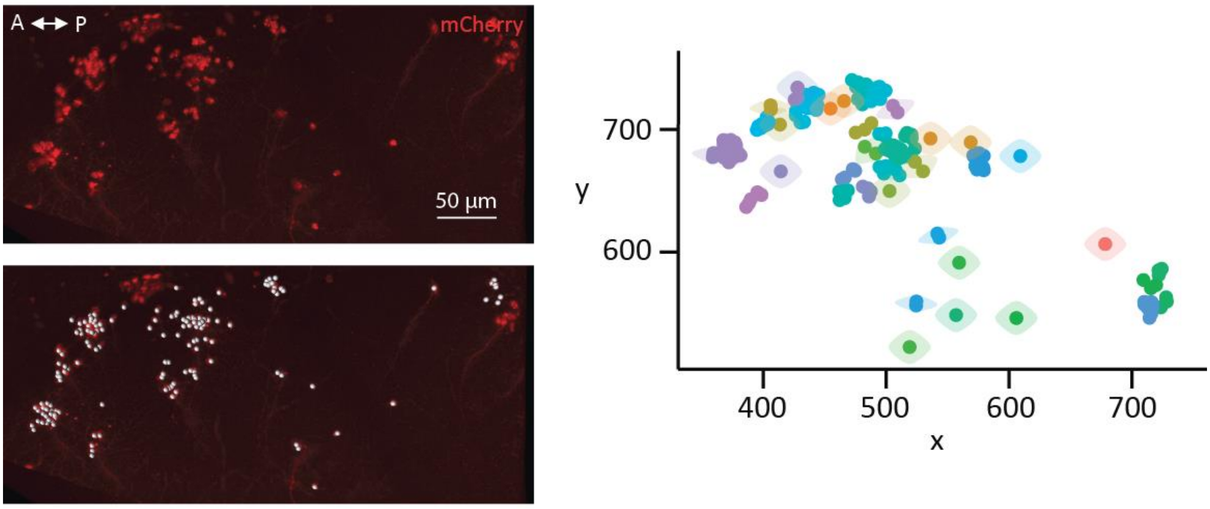
A ↔ P
mCherry
50 µm
700
600
y
400
500
600
700
x

125 dpi

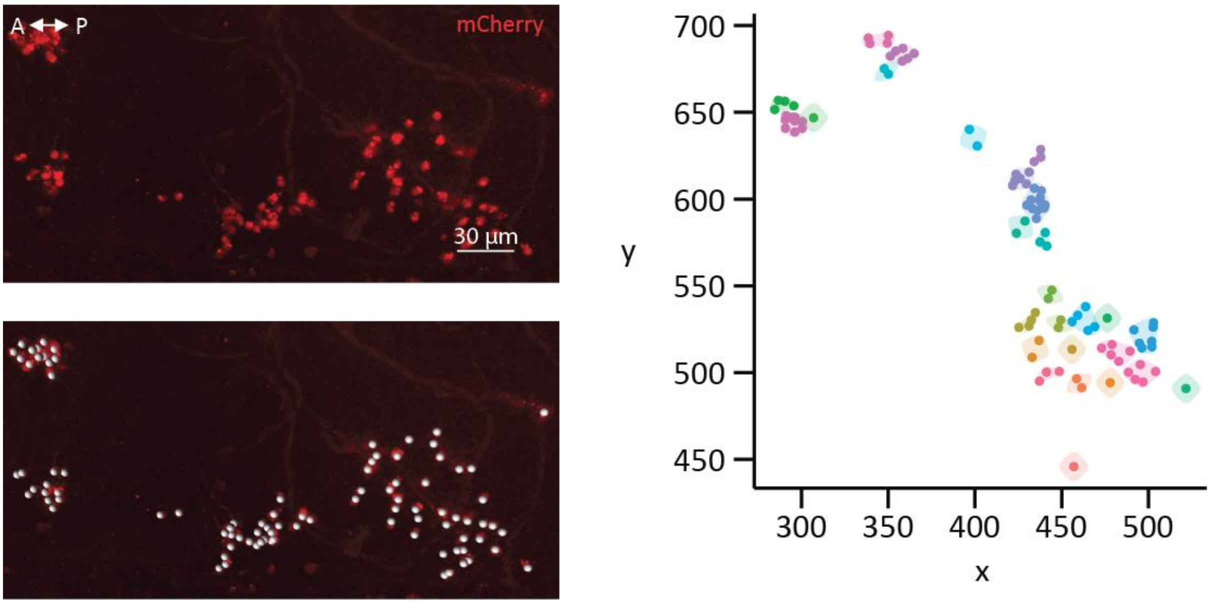

183 dpi

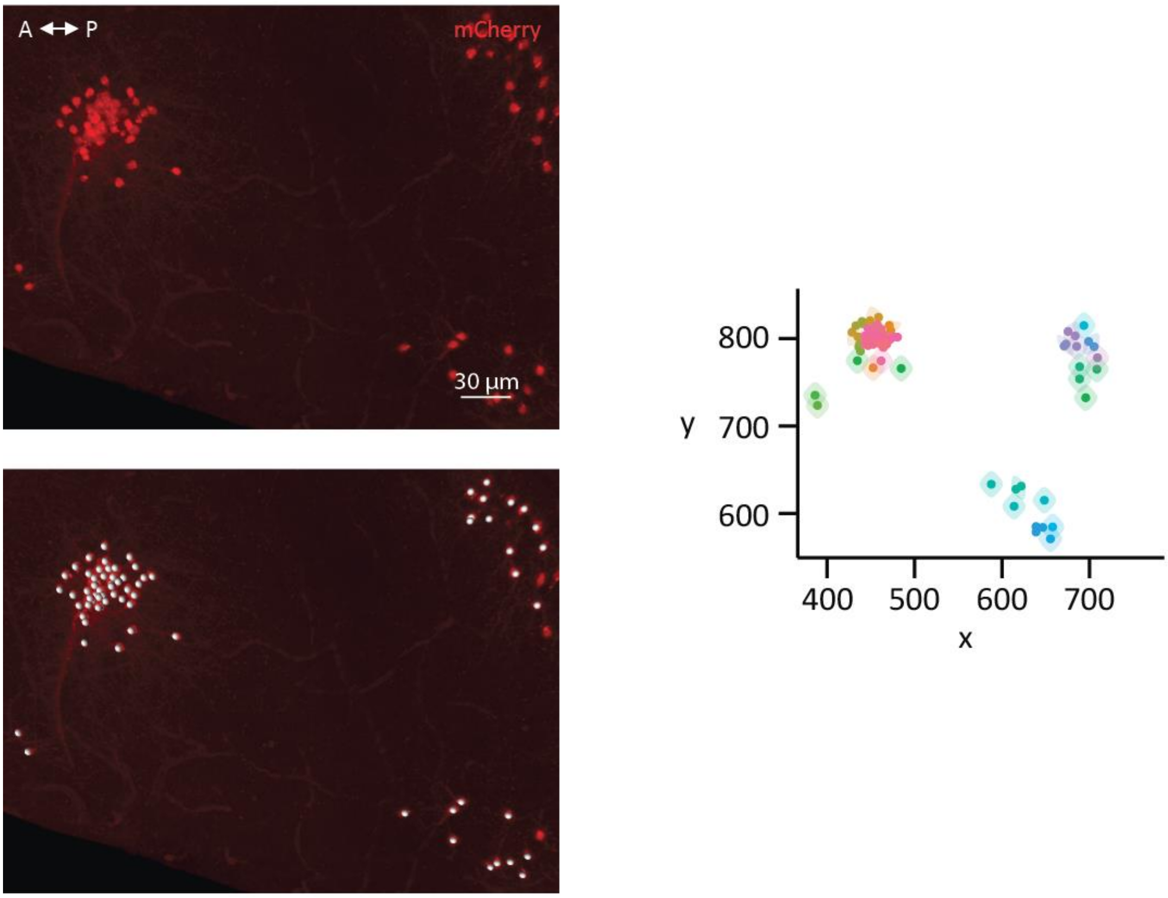

307 dpi

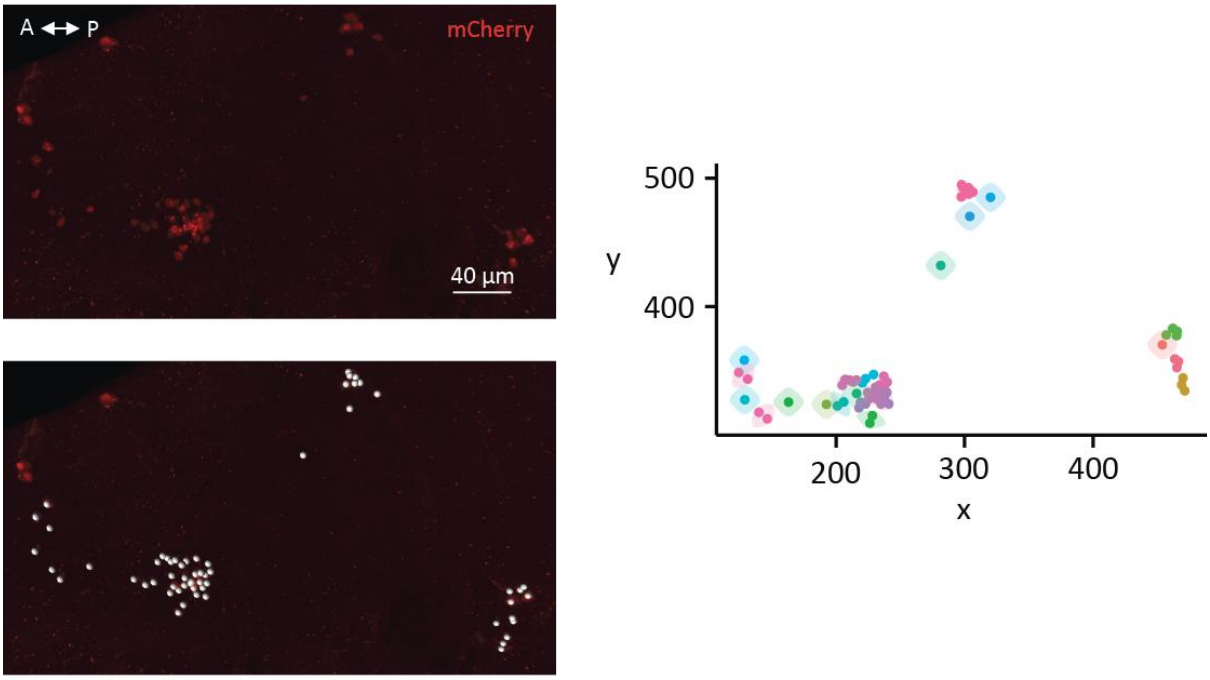

507 dpi

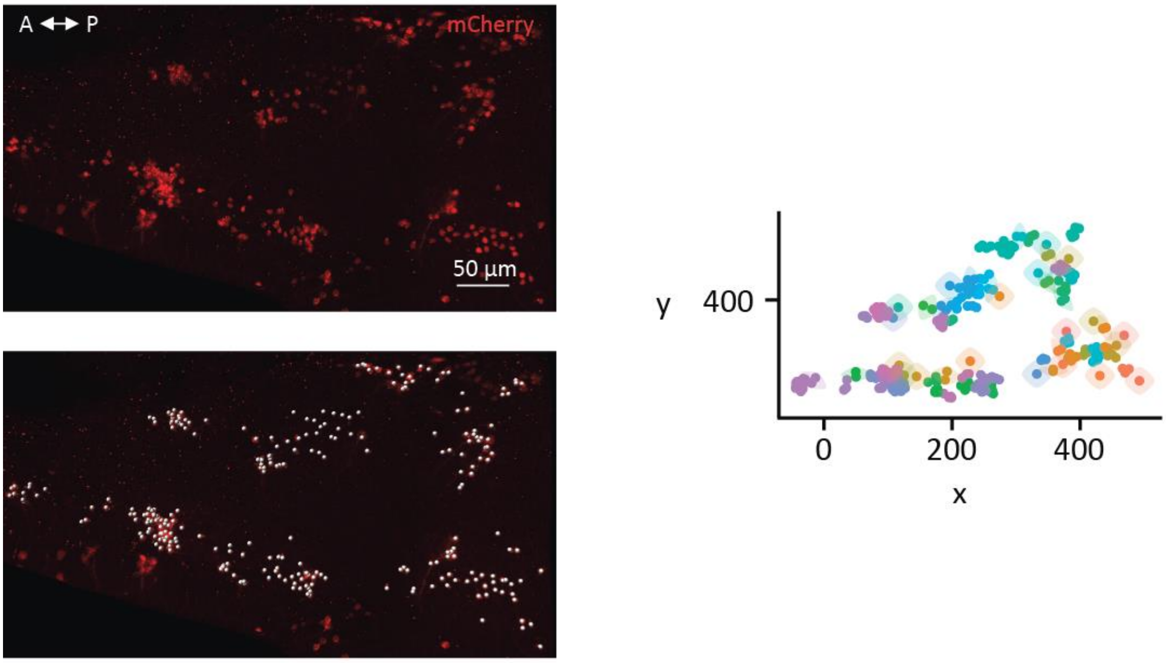

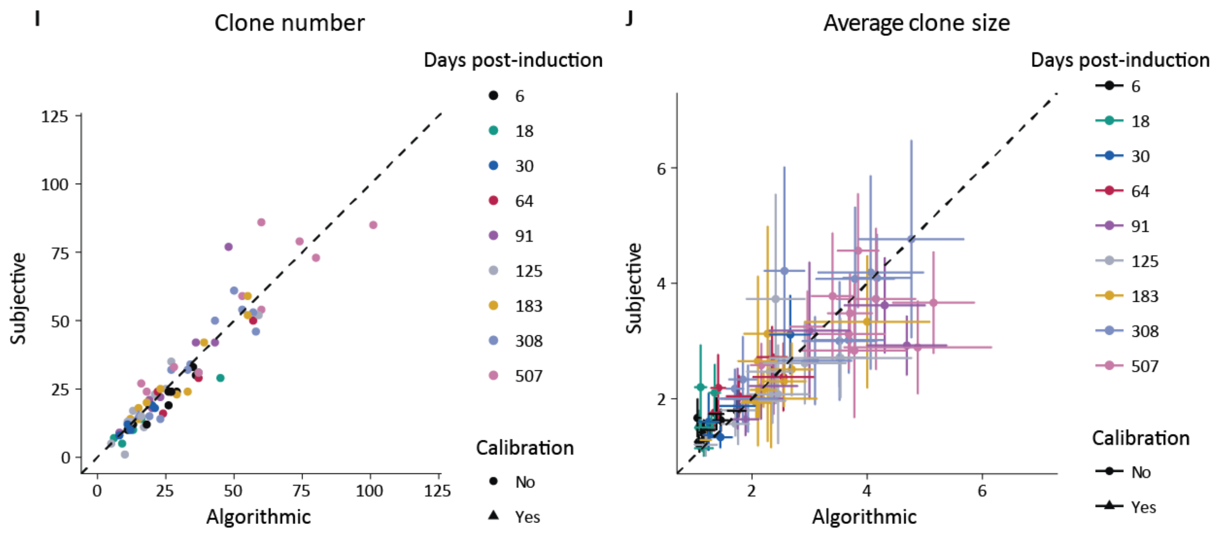

Fig. S4. Clonality assessment. (**A**) Dorsal view of the pallium at 6 dpi; mCherry$^+$ cell clusters comprising at least one Sox2$^+$ cells are highlighted with a white spot. (**B**) Cumulative probability distribution of the nearest neighbor distances (NNDs) between the Sox2$^+$ cell-containing clones at 6 dpi. n=6 brains. Error bars: s.e.m. (**C**) Left: Dorsal view (3D reconstruction) of a pallium immunostained for Sox2 and activated Caspase 3 (aCas3). The Dm domain analyzed is circumscribed by the red dashed line. Center: Enlargement of the region framed in green on the left. Right: Optical section along the plane defined by the dotted blue line on the central image. Note that the aCas3$^+$ cell is located under the ventricular area and does not express Sox2. (**D**) Quantification of the number of Sox2$^-$ and Sox2$^+$ cells displaying immunoreactivity for aCas3. aCas3 was detected in none of the Sox2$^+$ cells. n=7 brains (from 6 mpf zebrafish). (**E**) Average number of visually determined clones. One-way ANOVA: $F_{(8,37)}=2.76$, p=0.017; All pairwise comparisons: LSD test followed by Holm's adjustment;* p<0.05. Error bars: 95% CI. n= 6, 3, 3, 3, 4, 6, 7, 8 and 6 brains at 6, 18, 30, 64, 91, 125, 183, 307 and 507 dpi, respectively. (**F**) Same as (E) without the last time point. Note the apparent steadiness of the average number of visually determined clones after removing the last time point (507 dpi). One-way ANOVA: $F_{(7,32)}=1.66$, p=0.15. All pairwise comparisons: LSD test followed by Holm's adjustment; p>0.05 for all comparisons. n= 6, 3, 3, 3, 4, 6, 7 and 8 brains at 6, 18, 30, 64, 91, 125, 183 and 307, respectively. Error bars: 95% CI. (**G**) Number of clones determined by the clustering algorithm (black box and whisker plots). Box and whisker plots: the central bold bar and the upper and lower edges of the boxes represent respectively the mean and s.e.m. of the most likely clonal composition; the whiskers of the box correspond respectively to the 95% CIs of the smallest and biggest clustering that are still within the 95% CI of the most likely clustering (see supplementary text). They reflect the combined uncertainty stemming from the clonal reconstruction and the finite sample size. The mean ± s.e.m. of the visually determined number of clones is also given for comparison (green box). n= 6, 3, 3, 3, 4, 6, 7, 9 and 7 brains at 6, 18, 30, 64, 91, 125, 183, 307 and 507 dpi, respectively. (**H**) Representative examples of clustering for the different time points. A dorsal view of a three-dimensional reconstruction of a hemisphere is given on the left. The mCherry channel is displayed on the upper image to show the traced cells and the spots registering their coordinates are shown superimposed on the lower image. A two-dimensional map corresponding to a planar (xy) projection of the traced cells is given

on the right. Axes correspond to the x and y coordinates (in μm) of the 2D projection of the cell centers. The cells belonging to the most likely clonal partitions determined by statistical inference (i.e. inferred clones) are embedded in the same shaded area. Note that a number of different clones may share the same color owing to the limited number of colors available for display. (**I**) Scatter plot showing the correlation between the numbers of clones determined by the algorithm (x-axis) and visually (y-axis). (**J**) Same plot for the average size of clones. (**I** and **J**) The different time points of the clonal analysis are represented with different colors. n= 6, 3, 3, 3, 4, 6, 7, 9 and 7 brains at 6, 18, 30, 64, 91, 125, 183, 307 and 507 dpi, respectively.

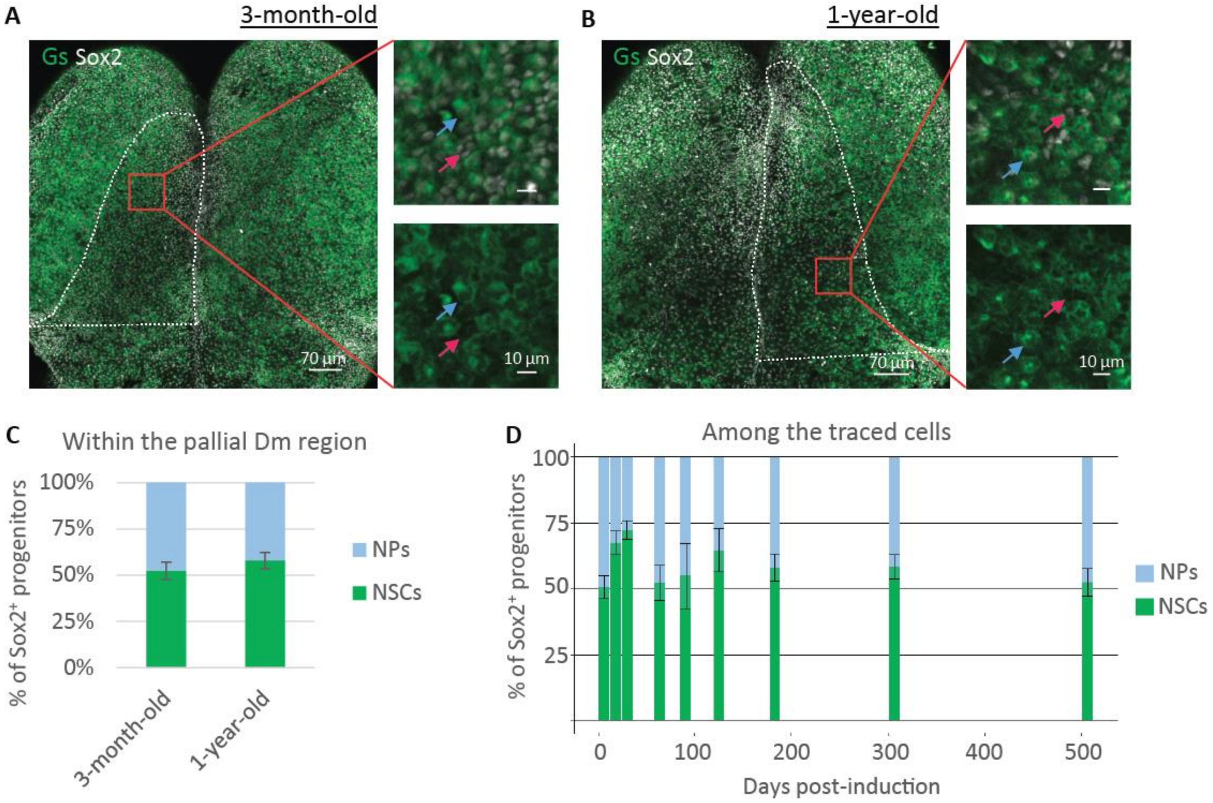

Fig. S5. The behavior of Sox2$^+$ progenitors reads out that of NSCs. (**A**-**B**) Dorsal view of 3 mpf (**A**) and 12 mpf (**B**) pallia immunostained for Gs and Sox2. The boxed areas are enlarged on the right to display Gs$^+$/Sox2$^+$ NSCs (blue arrow) and Gs$^-$/Sox2$^+$ NPs (pink arrow). (**C**) Quantification of the relative proportions of NSCs and NPs in the analyzed Dm region at 3 mpf and 12 mpf. p=0.43, unpaired t-test. Error bars: s.e.m. n=4 brains for both ages. (**D**) Evolution over time of the ratio of NSCs to NPs among the traced population of progenitors. One-way ANOVA: $F_{(8,37)}$=1.04, p=0.42; pairwise comparison: LSD test followed by Holm's adjustment. Error bars: s.e.m. n= 6, 3, 3, 3, 4, 6, 7, 8 and 6 brains at 6, 18, 30, 64, 91, 125, 183, 307 and 507 dpi, respectively.

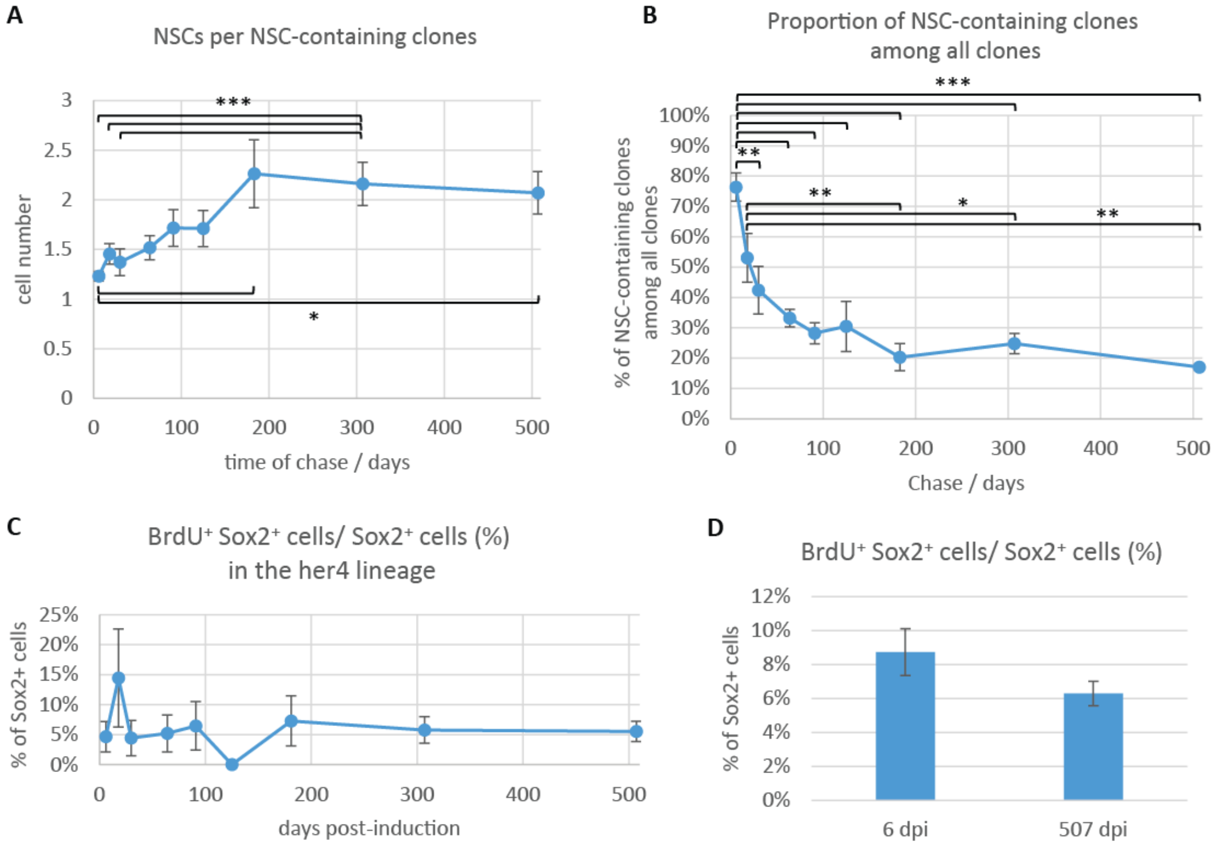

Fig. S6. Hierarchical organization of pallial NSCs. (**A** and **B**) Clonal dynamics using a visual determination of clones. (**A**) Time evolution of the number of NSCs (approximated by $Sox2^+$ cells) per NSC-containing clone. Kruskal-Wallis test: p=0.0042; all pairwise comparisons: Behrens Fisher tests. * p< 0.05, *** p<0.001. Error bars: s.e.m. (**B**) Evolution over time of the proportion of NSC-containing clones (i.e. clones maintaining at least one $Sox2^+$ cell) among all clones. The proportion of reservoir NSCs among the traced NSCs is given by the value of the plateau (~ 20%). One-way ANOVA: $F_{(8,37)}=14.82$, p<0.001; all pairwise comparisons: LSD test followed by Holm's adjustment. * p< 0.05, ** p<0.01, *** p<0.001. Error bars: s.e.m. (**C**) Evolution over time of the proportion of proliferating NSCs (i.e. $Sox2^+$ cells) within the lineage. Kruskal-Wallis test: p=0.49; pairwise comparisons: p>0.05 for all comparisons (Behrens Fisher tests). Error bars: s.e.m. (**D**) Comparison of the proportion of proliferating NSCs (i.e. $Sox2^+$ cells) in the analyzed pallial area between the first and the last time point of the chase. Welch's t-test assuming unequal variances: p=0.15. n=6 brains for each conditions. (**A-C**) n= 6, 3, 3, 3, 4, 6, 7, 8 and 6 brains at 6, 18, 30, 64, 91, 125, 183, 307 and 507 dpi, respectively.

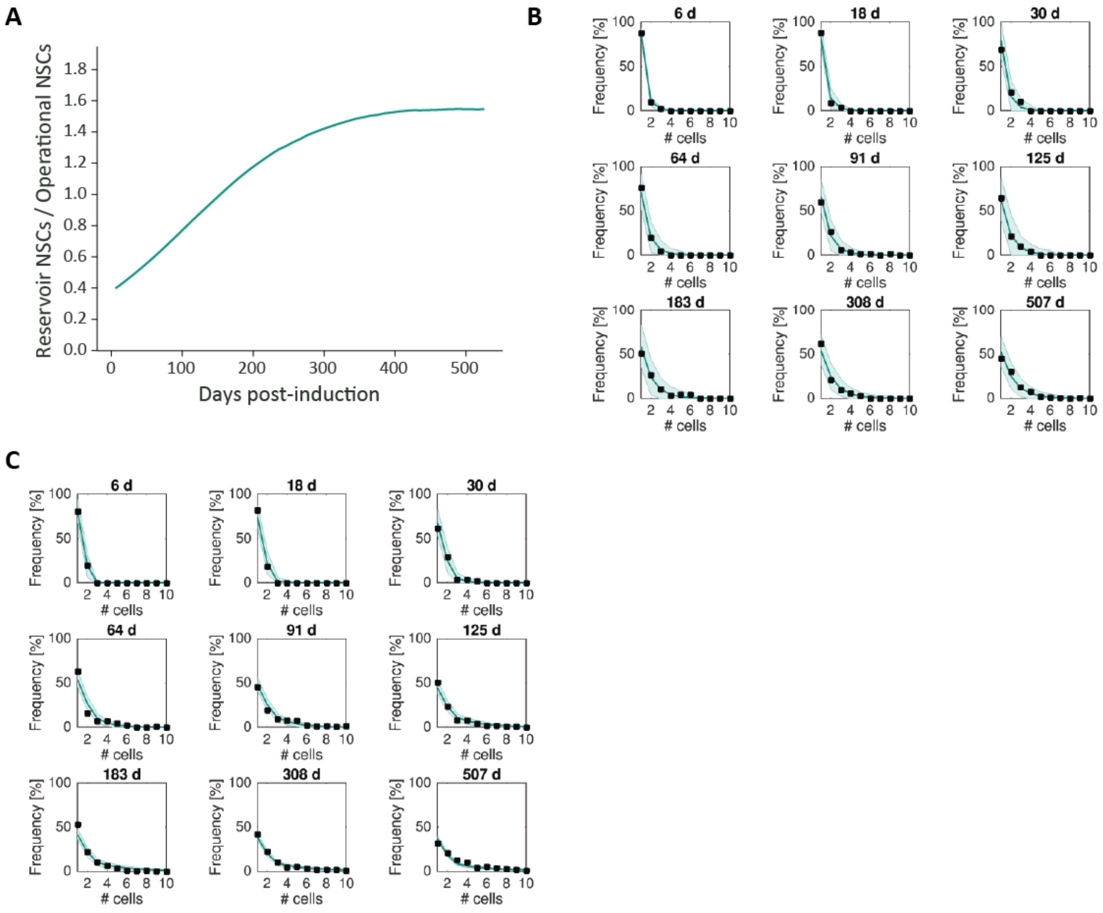

Fig. S7. Clone size distributions. (**A**) Time evolution of the ratio of reservoir to operational NSCs as inferred from the modeling. The experimental ratio at 6 dpi (0.4) was used to initialize the modeling. Because induction was likely biased towards operational NSCs, this ratio progressively increase from 0.4 at 6 dpi until reaching a steady state value of 1.57, which correspond to the putative actual ratio within the *her4*$^{+}$ NSC lineage. (**B**) Inverse cumulative distribution of the NSC content of the clones, indicating the chance of finding a clone with more than a given number of NSCs. (**C**) Inverse cumulative distribution of the neuronal content of the clones, indicating the chance of finding a clone with more than a given number of neurons. (**B** and **C**) Black squares represent the means from the experimental data. Green curves and light green shaded areas correspond to the model predictions and their 95 CIs, respectively.

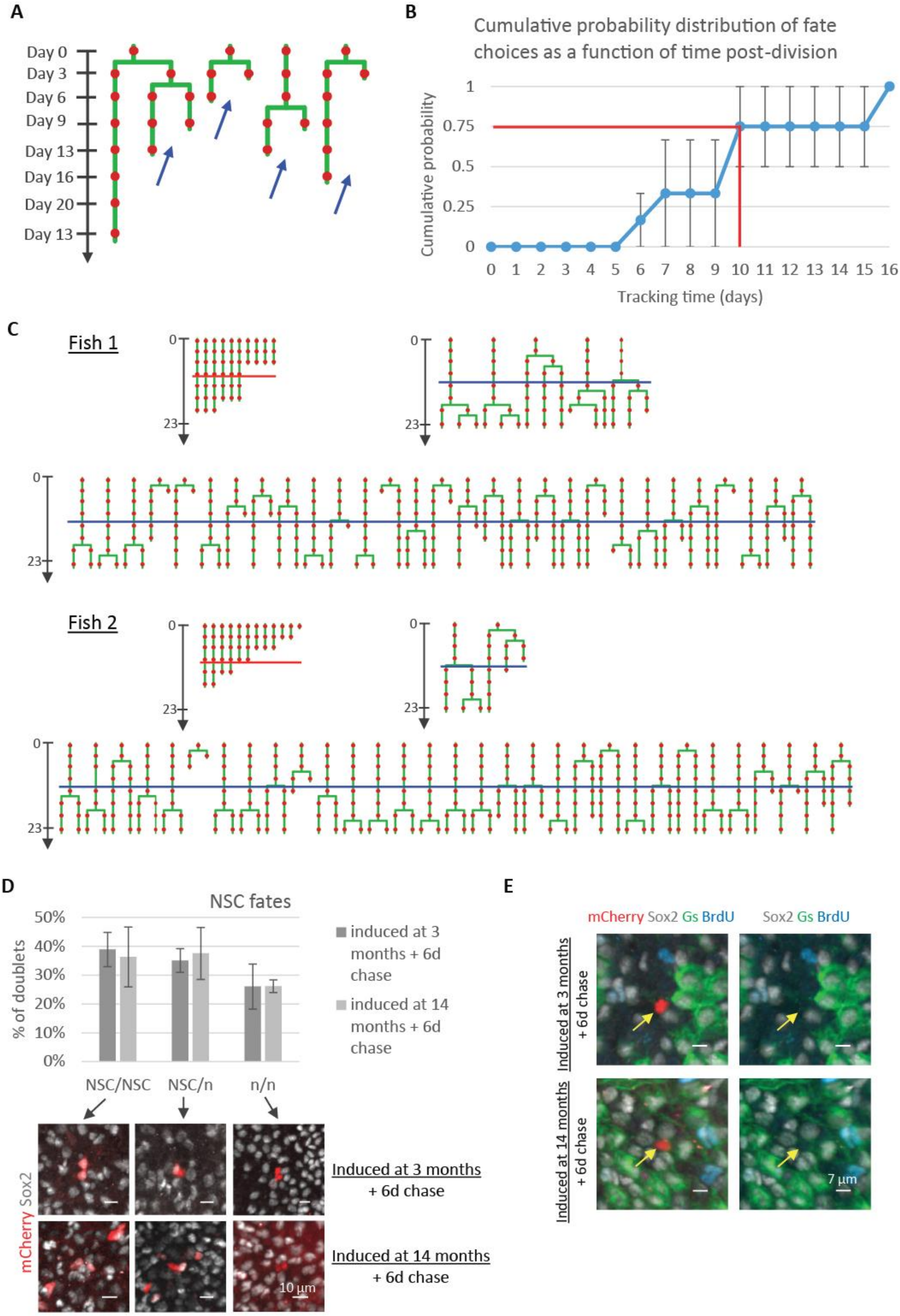
A
Day 0
Day 3
Day 6
Day 9
Day 13
Day 16
Day 20
Day 13
B
Cumulative probability distribution of fate choices as a function of time post-division
Cumulative probability
Tracking time (days)
C
Fish 1
Fish 2
0
23
D
NSC fates
% of doublets
induced at 3 months + 6d chase
induced at 14 months + 6d chase
NSC/NSC
NSC/n
n/n
mCherry Sox2
Induced at 3 months + 6d chase
Induced at 14 months + 6d chase
10 µm
E
mCherry Sox2 Gs BrdU
Sox2 Gs BrdU
Induced at 3 months + 6d chase
Induced at 14 months + 6d chase
7 µm

Fig. S8. Analysis of NSC short-term behavior. (**A**) Example of tracks harboring a symmetric neurogenic division (blue arrows - days of imaging sessions are indicated on the left). (**B**) Cumulative probability distribution of the time (in days) elapsed between a NSC symmetric neurogenic division and the moment when its last daughter cell lost *gfap*:dTomato expression. 75% of fate choices were apparent before ten days of tracking (red lines). The time of division was estimated by the average time between the last time point before division and the first time point following division. A similar strategy was used to determine the time of differentiation. Based on 3 and 2 neurogenic divisions from n=2 brains. Error bars: s.e.m. (**C**) Illustration of all the active tracks (i.e. comprising either a division event or a direct neuronal differentiation) in the Dm territory of interest (n=2). These are a total of 44 and 45 NSCs from 320 and 373 NSCs imaged, respectively (i.e. 276 and 328 NSCs remained quiescent for the entire duration of the recording). For each fish: top left: direct differentiation tracks; top right: 2 division-tracks; bottom: 1 division-tracks. We only analyzed the outcome of divisions occurring at least three time points (i.e. about 10 days – blue bar) before the end of the experiment, and only scored direct differentiation events when taking place after at least four recorded time points (red bar). (**D**) Two-celled clones (doublets) recovered at 6 dpi (Fig. S2) were used to infer NSC fate upon division (“n”: neuron, i.e. $Sox2^-$ cell). The graphic shows the relative proportions of the different types of NSC divisions inferred from the doublets. Illustrations of doublets of each type are given below the graphic. Inductions were performed in both 3-month-old and 14-month-old fish and revealed no change in NSC fates with age. Only brains harboring at least 6 doublets were analyzed. 3-month-old fish: n=5 brains; 14-month-old fish: n= 3. (**E**) Example of single-celled neuronal clones (i.e. lacking Sox2 expression), expected to arise from the direct differentiation of a NSC into a neuron.

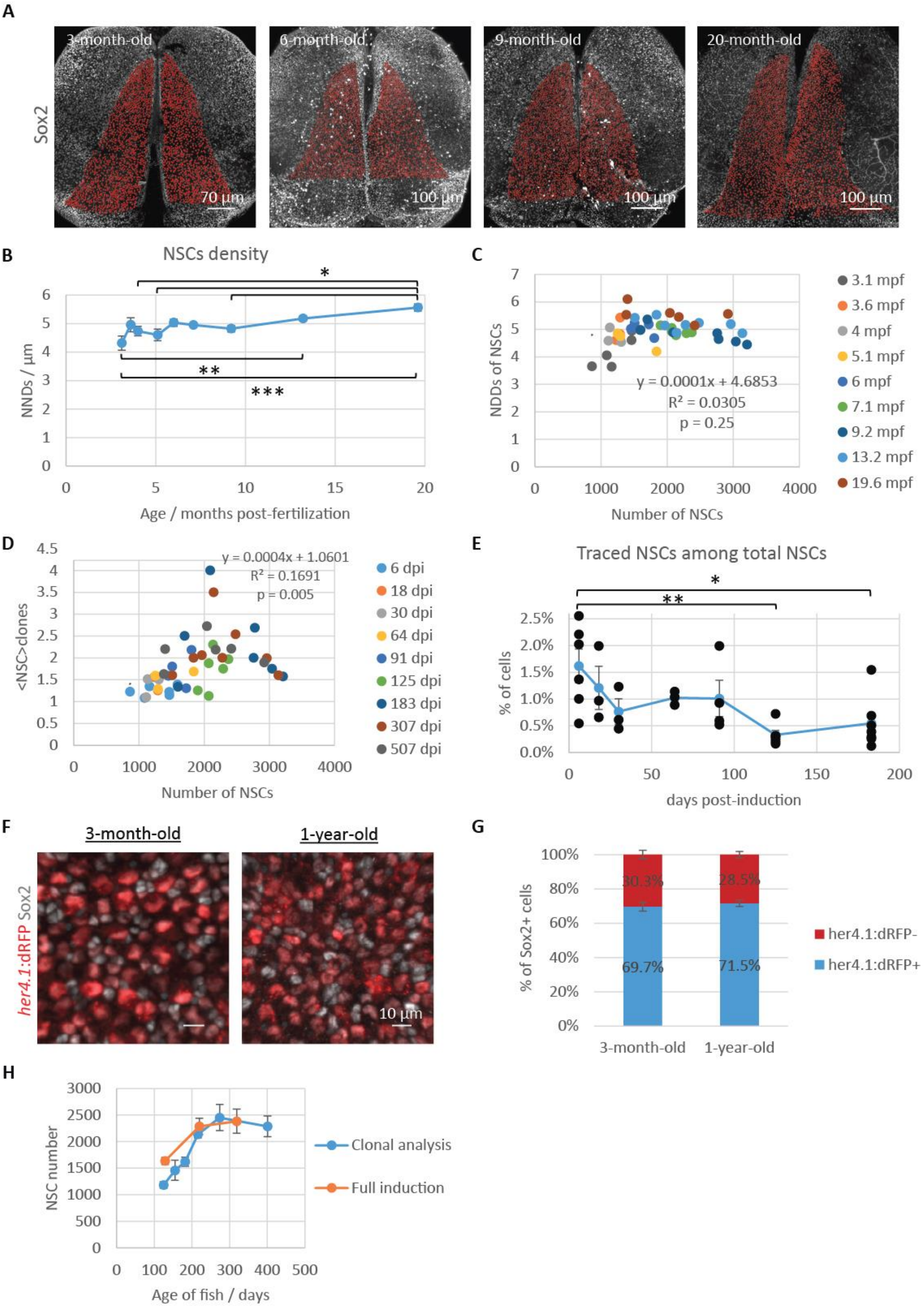

A
3-month-old
6-month-old
9-month-old
20-month-old
Sox2
70 µm
100 µm
100 µm
100 µm
B
NSCs density
NNDs / µm
Age / months post-fertilization
C
NDDs of NSCs
Number of NSCs
y = 0.0001x + 4.6853
R² = 0.0305
p = 0.25
3.1 mpf
3.6 mpf
4 mpf
5.1 mpf
6 mpf
7.1 mpf
9.2 mpf
13.2 mpf
19.6 mpf
D
<NSC>clones
Number of NSCs
y = 0.0004x + 1.0601
R² = 0.1691
p = 0.005
6 dpi
18 dpi
30 dpi
64 dpi
91 dpi
125 dpi
183 dpi
307 dpi
507 dpi
E
Traced NSCs among total NSCs
% of cells
days post-induction
F
3-month-old
1-year-old
her4.1:dRFP Sox2
10 µm
G
% of Sox2+ cells
30.3%
69.7%
28.5%
71.5%
3-month-old
1-year-old
her4.1:dRFP-
her4.1:dRFP+
H
NSC number
Age of fish / days
Clonal analysis
Full induction

Fig. S9. Ongoing production of NSCs by an upstream progenitor source. (**A**) Dorsal view of three-dimensional reconstructions of pallia immunostained for Sox2 at different ages. All NSCs (orange dots) were counted in the Dm region of interest. (**B**) Evolution of NSC nearest neighbor distances (NNDs) with advancing age. One-way ANOVA: $F_{(8,37)}=6.09$, $p<0.001$; all pairwise comparisons: LSD test followed by Holm's adjustment. * $p< 0.05$, ** $p<0.01$, ***$p<0.001$. Error bars: s.e.m. (**C**) Scatter plot showing the complete absence of correlation between the number of NSCs ($Sox2^+$ cells) and their density as assessed by their NNDs. Cell numbers are per hemisphere. (**B**-**C**) n= 6, 3, 3, 3, 4, 6, 7, 8 and 6 brains at 3.1, 3.6, 4, 5.1, 6, 7.1, 9.2, 13.2 and 19.6 mpf, respectively. (**D**) Scatter plot presenting the relationship between the number of NSCs and the NSC content of the NSC-containing clones. The minor correlation (r = 0.41) between both statistics is likely fortuitous and appears insufficient to explain the expansion of the NSC population by the growth of the NSC content of the NSC-containing clones. n= 6, 3, 3, 3, 4, 6, 7, 8 and 6 brains at 6, 18, 30, 64, 91, 125, 183, 307 and 507 dpi, respectively. Cell numbers are per hemisphere. (**E**) Evolution of the proportion of traced ($mcherry^+$) NSCs among all NSCs during their period of expansion. One-way ANOVA: $F_{(8,37)}=3.48$, $p<0.01$; all pairwise comparisons: LSD test followed by Holm's adjustment. * $p< 0.05$, ** $p<0.01$. Error bars: s.e.m. n= 6, 3, 3, 3, 4, 6 and 7 brains at 6, 18, 30, 64, 91, 125 and 183 dpi, respectively. (**F**) Dorsal views (close-ups) of the analyzed pallial region of 3-month-old and 1-year-old *her4.1*:dRFP transgenic fish immunostained for Sox2. (**G**) Respective proportion of *her4.1*:$dRFP^+$ and *her4.1*:$dRFP^-$ cells among the total $Sox2^+$ population of cells. Unpaired t-test: p=0.59. n=4 brains for both ages. (**H**) Comparison of the number of pallial NSCs present in fish of different ages between the clonal analysis and the full induction experiment. Error bars: s.e.m. Clonal analysis: n= 3, 3, 4, 6, 7 and 8 brains at 125, 155, 182, 216, 274 and 401 days old, respectively. Full induction: n= 5, 6, and 5 brains at 129, 220 and 319 days old, respectively.

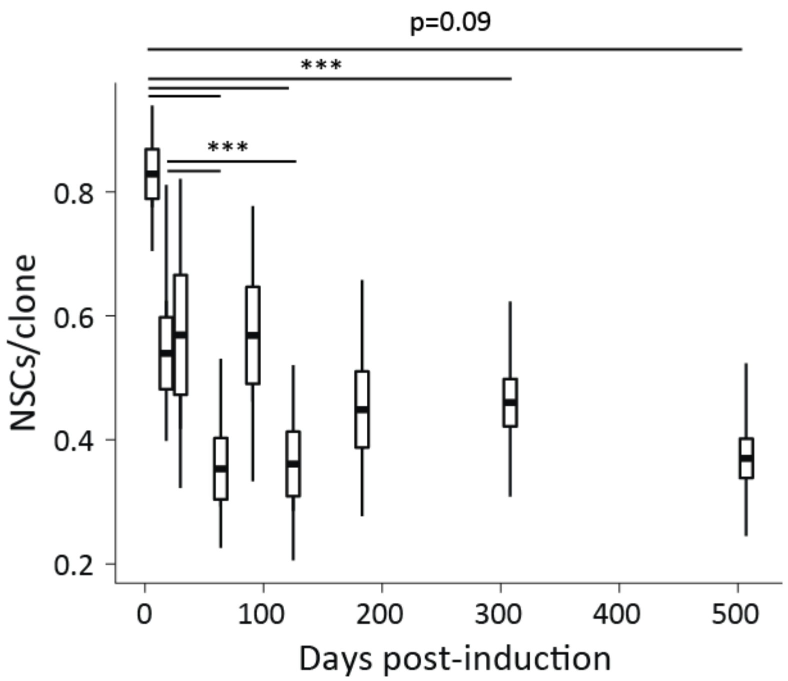

Fig. S10. Average number of NSCs per clone. Box and whisker plots: experimental data. The central bold bar and the upper and lower edges of the boxes represent respectively the mean and s.e.m. of the most likely clonal composition; the whiskers of the box correspond respectively to the 95% CIs of the most extreme clonal assignments still in agreement with clonality (see supplementary text). They reflect the combined uncertainty stemming from the clonal reconstruction and the finite sample size. Kruskal-Wallis test: $p=0.022$; all pairwise comparisons: Behrens Fisher tests. ** $p<0.001$. n= 6, 3, 3, 3, 4, 6, 7, 9 and 7 brains at 6, 18, 30, 64, 91, 125, 183, 307 and 507 dpi, respectively.

Data file S1 (Microsoft Excel format). Excel spreadsheet containing the results of the statistical tests corresponding to Figure 6
